# A genomic analysis reveals the diversity of cellulosome displaying bacteria

**DOI:** 10.3389/fmicb.2024.1473396

**Published:** 2024-10-30

**Authors:** Christine M. Minor, Allen Takayesu, Sung Min Ha, Lukasz Salwinski, Michael R. Sawaya, Matteo Pellegrini, Robert T. Clubb

**Affiliations:** ^1^Department of Chemistry and Biochemistry, University of California, Los Angeles, Los Angeles, CA, United States; ^2^UCLA-DOE Institute of Genomics and Proteomics, University of California, Los Angeles, Los Angeles, CA, United States; ^3^Department of Integrative Biology and Physiology, University of California, Los Angeles, Los Angeles, CA, United States; ^4^Department of Molecular, Cell, and Developmental Biology, University of California, Los Angeles, Los Angeles, CA, United States; ^5^Molecular Biology Institute, University of California, Los Angeles, Los Angeles, CA, United States

**Keywords:** Cellulosome, biomass, lignocellulose, comparative genomics, cohesin, dockerin, AlphaFold2

## Abstract

**Introduction:**

Several species of cellulolytic bacteria display cellulosomes, massive multi-cellulase containing complexes that degrade lignocellulosic plant biomass (LCB). A greater understanding of cellulosome structure and enzyme content could facilitate the development of new microbial-based methods to produce renewable chemicals and materials.

**Methods:**

To identify novel cellulosome-displaying microbes we searched 305,693 sequenced bacterial genomes for genes encoding cellulosome proteins; dockerin-fused glycohydrolases (DocGHs) and cohesin domain containing scaffoldins.

**Results and discussion:**

This analysis identified 33 bacterial species with the genomic capacity to produce cellulosomes, including 10 species not previously reported to produce these complexes, such as *Acetivibrio mesophilus*. Cellulosome-producing bacteria primarily originate from the *Acetivibrio, Ruminococcus, Ruminiclostridium*, and *Clostridium* genera. A rigorous analysis of their enzyme, scaffoldin, dockerin, and cohesin content reveals phylogenetically conserved features. Based on the presence of a high number of genes encoding both scaffoldins and dockerin-fused GHs, the cellulosomes in *Acetivibrio* and *Ruminococcus* bacteria possess complex architectures that are populated with a large number of distinct LCB degrading GH enzymes. Their complex cellulosomes are distinguishable by their mechanism of attachment to the cell wall, the structures of their primary scaffoldins, and by how they are transcriptionally regulated. In contrast, bacteria in the *Ruminiclostridium* and *Clostridium* genera produce ‘simple’ cellulosomes that are constructed from only a few types of scaffoldins that based on their distinct complement of GH enzymes are predicted to exhibit high and low cellulolytic activity, respectively. Collectively, the results of this study reveal conserved and divergent architectural features in bacterial cellulosomes that could be useful in guiding ongoing efforts to harness their cellulolytic activities for bio-based chemical and materials production.

## Introduction

Lignocellulosic plant biomass (LCB) is the largest source of carbon in the biosphere and a promising feedstock for the production of renewable materials, biofuels, and chemicals (Bar-On et al., 2018). LCB’s utility is limited by its recalcitrance to hydrolysis which makes it costly to degrade at an industrial scale (Chundawat et al., 2011; Liu et al., 2021). LCB consists of crystalline cellulose (32–47% dry weight) and hemicellulose (19–27%) encased within a web of cross-linked monolignols that forms lignin (5–24%; Shukla et al., 2023). Several species of highly cellulolytic anaerobic bacteria have garnered significant interest as potential tools to efficiently deconstruct LCB into its component sugars for use in bio-commodity production (Wang et al., 2024). These microbes display massive multi-cellulase containing complexes called cellulosomes that degrade LCB’s cellulose and hemicellulose components (Dassa et al., 2017; Artzi et al., 2017; Gilbert, 2007; Bayer et al., 2004; Smith and Bayer, 2013; Doi et al., 2003). A deeper understanding of the structural diversity of these complexes in bacteria could facilitate their usage in industrial applications.

Cellulosomes were first discovered in *Acetivibrio thermocellus* (formerly known as *Clostridium thermocellum* and *Hungateiclostridium thermocellum*) as a “cellulose-binding factor,” and subsequently have been found in a range of anaerobic eubacteria (Artzi et al., 2017; Lamed et al., 1983). These multi-cellulase complexes are constructed from scaffolding proteins (called scaffoldins) that coordinate the binding of an array of glycoside hydrolases (GH) (Dassa et al., 2017; Artzi et al., 2017; Gilbert, 2007; Bayer et al., 2004; Smith and Bayer, 2013). Each scaffoldin contains one or more cohesin domains that bind noncovalently to dockerin domains that are genetically fused to the GHs (called DocGH enzymes). In addition, scaffoldins can harbor carbohydrate binding module (CBM) domains, cell wall (e.g., S-layer homology (SLH) domains) binding modules, and dockerin domains that interact with other scaffoldins on the cell surface. For example, the ScaA scaffoldin in *A. thermocellus* contains multiple cohesin domains that bind DocGH enzymes, an internal CBM type-3 domain (CBM3) that binds cellulose, and a C-terminal dockerin domain that enables it to associate with a series of cell wall associated scaffoldins (ScaB, ScaC, ScaD, and ScaF) (Hong et al., 2014). Three major types of GHs function synergistically to degrade cellulose: endoglucanases, exoglucanases, and β-glucosidases (Bhardwaj et al., 2021; Sharma et al., 2016). Endoglucanases hydrolyze internal β-(1,4)-glycosidic bonds in cellulose (e.g., GH7, GH12), creating reducing and non-reducing ends that are further hydrolyzed by exoglucanases (e.g., GH5, GH9). The resulting cellodextrin carbohydrate oligomers are then degraded into glucose by β-glucosidases. The carbohydrate substrate specificities of cellulosomal DocGH enzymes vary, but members of the GH5, GH10, GH11, GH43, and GH48 families are frequently present in cellulosome producing bacteria (Artzi et al., 2017). Other types of carbohydrate active enzymes (CAZymes) are also fused to dockerin domains enabling their incorporation into cellulosomes, including polysaccharide lyases (PLs), and carbohydrate esterases (CEs). Collectively, the diversity of DocGH enzymes, CAZymes, and CBM modules within the cellulosome enable bacteria to degrade LCB more efficiently than microbes that simply secrete GHs, because enzyme colocalization by the cellulosome promotes enzyme–enzyme synergy, enzyme-proximity enhancement, and cellulose-enzyme-microbe interactions (Lu et al., 2006; Barba-Cedillo and Montanier, 2023; Smith et al., 2017).

**Figure 1:**
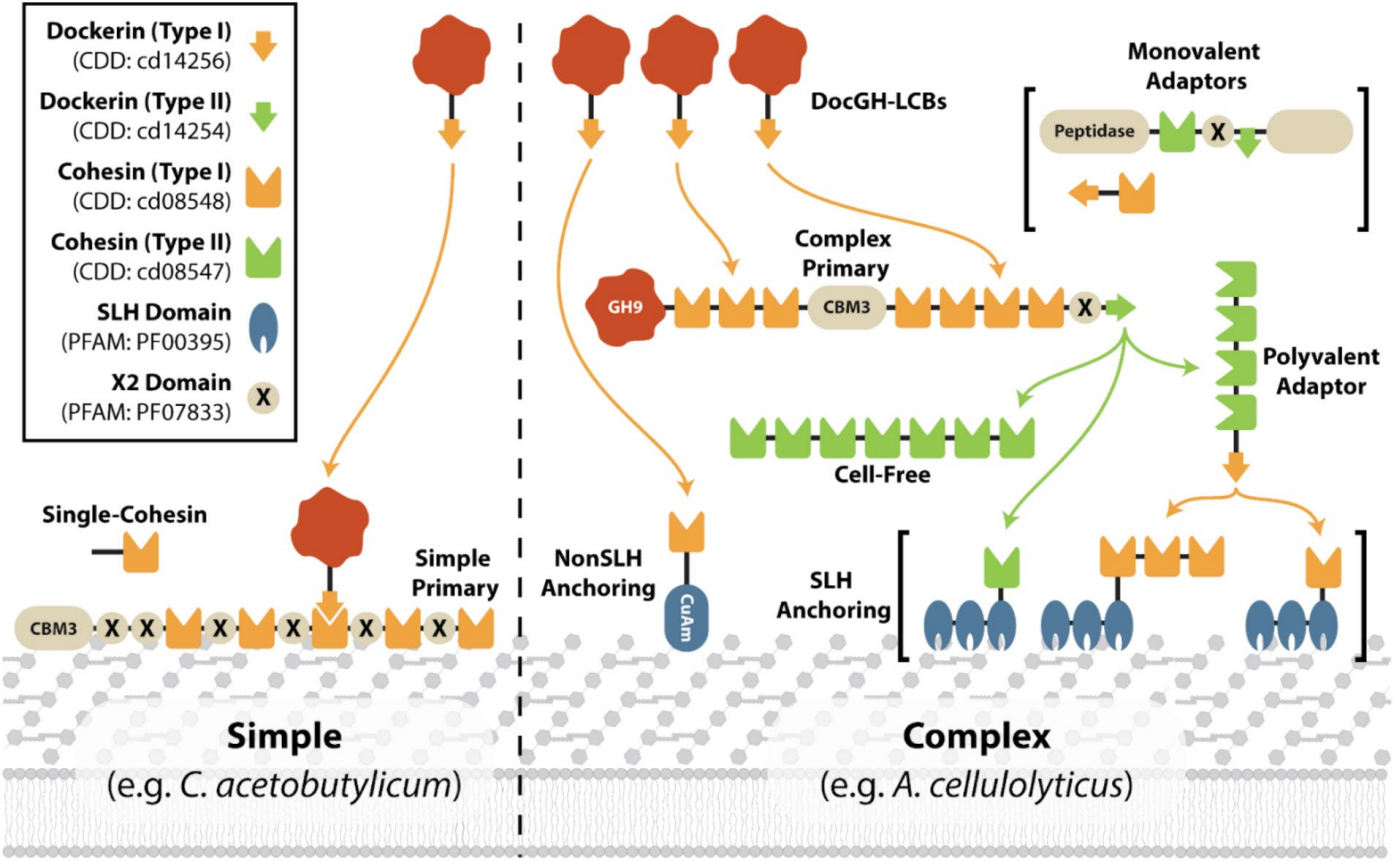
Representative scaffoldin compositions in simple and complex cellulosomes. Cartoon showing the different types of scaffoldins that are found in either simple (e.g., *C. acetobutylicum*; Nölling et al., 2001) or complex (e.g., *A. cellulolyticus*; Hamberg et al., 2014; Dassa et al., 2012) cellulosomes. Simple cellulosomes typically contain a simple primary scaffoldin and a small number of accessory scaffoldins. Complex cellulosomes contain a multi-cohesin complex primary scaffoldin with an associated anchoring scaffoldin, either a SLH or nonSLH-anchoring scaffoldin. Many complex cellulosomes also contain adaptor scaffoldins (polyvalent or monovalent) and cell-free scaffoldins that are presumably secreted. An example of a cohesin:DocGH interaction is shown on the left.

Several species of mesophilic and thermophilic anaerobic bacteria display cellulosomes that vary in their complexity and composition. These include complex, simple, and cell-free cellulosomes that differ in both the number and types of cohesin-containing scaffoldins they possess (Dassa et al., 2017; Artzi et al., 2017; Bule et al., 2018; Bae et al., 2013). Three types of cohesin (Coh1, Coh2, and Coh3) and dockerin (Doc1, Doc2, and Doc3) domains have been identified based on their primary sequences (Bayer et al., 2004). Biochemical experiments have shown that these domains typically interact with one another in a species- and type-specific manner (e.g., Doc1 binds to Coh1 domains, but not with Coh2 or Coh3 domains within the same species; Leibovitz and Béguin, 1996; Pagès et al., 1997), however, there are several exceptions (Hamberg et al., 2014; Phitsuwan et al., 2019; Artzi et al., 2014). It has also been noted that there are two distinct conformations Doc1 modules can bind to Coh1 modules, further expanding the possible cellulosome architectures (Carvalho et al., 2007; Cameron et al., 2015). Complex cellulosomes, typified by the one present in *Acetivibrio cellulolyticus*, contain a primary scaffoldin that harbors several Coh1 modules for DocGH binding and a C-terminal Doc2 that enables it to interact with Coh2 modules presented in cell wall associated anchoring scaffoldins (Figure 1, right; Brás et al., 2016). In many cases, microbes containing this type of primary scaffoldin also possess adaptor scaffoldins that harbor both cohesin and dockerin domains that are believed to expand both the number and types of DocGH proteins that are incorporated into the cellulosome [e.g., *Acetivibrio clariflavus* (Artzi et al., 2014), *Pseudobacteroides cellulosolvens* (Zhivin et al., 2017), *A. cellulolyticus* (Dassa et al., 2012), and *Acetivibrio alkalicellulosi* (Phitsuwan et al., 2019)]. Other bacterial species typified by *Clostridium acetobutylicum* produce simple cellulosomes that contain a singular multi-cohesin primary scaffoldin that houses an N-terminal CBM3 and interspersed X2 domains (e.g., *Clostridium cellulovorans, Ruminiclostridium cellulolyticum, Clostridium josui*; Figure 1, left; Dassa et al., 2017). The mechanism of cell surface attachment by simple cellulosomes is poorly understood but is likely mediated by the N-terminal CBM3 present on their primary scaffoldins as seen for *R. cellulolyticum* (Tao et al., 2022). Lastly, many microbes possess cell-free cellulosomes composed of multi-cohesin scaffoldins bound with DocGH enzymes which are secreted into the environment to degrade LCB (e.g., *A. clariflavus*) (Artzi et al., 2014; Raman et al., 2009).

**Figure 2:**
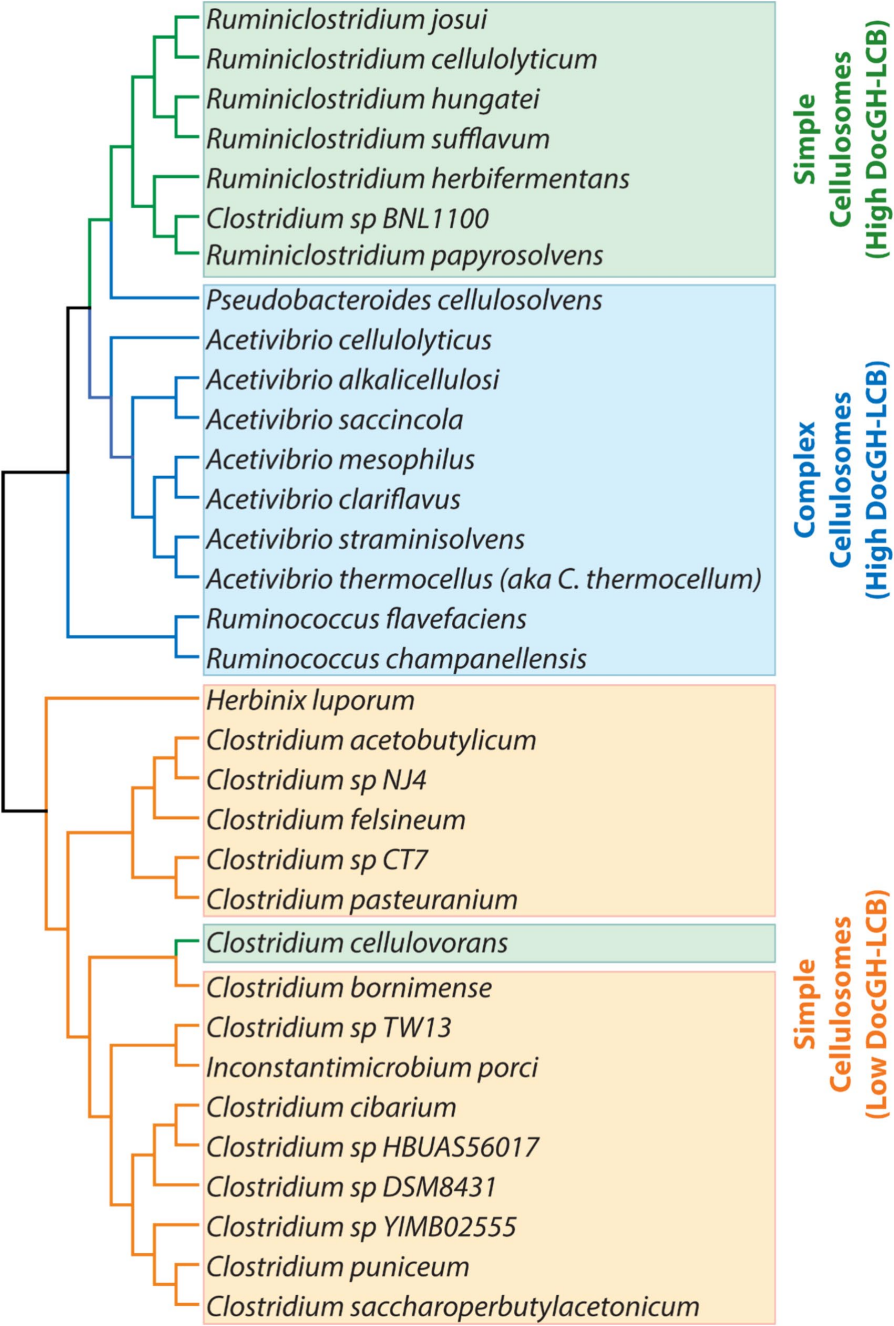
Taxonomic diversity of cellulosome producing bacteria. Phylogenetic tree of the bacteria listed in Tables 1, 2 (excluding the “scaffolding-containing” organisms). Bacteria are colored according to their predicted cellulosome type and number of genes encoding DocGH-LCB enzymes: Complex cellulosomes with high numbers of DocGH-LCB genes (blue); Simple cellulosome producing bacteria with high DocGH-LCB gene counts (green); Simple cellulosome producing bacteria with a low number of DocGH-LCB genes (orange). 16S rRNA sequences were aligned with ClustalW and the tree was constructed using MEGA11.

While a number of reviews have been written describing cellulosomes (Dassa et al., 2017; Artzi et al., 2017; Bae et al., 2013; Fontes and Gilbert, 2010), to the best of our knowledge, a systematic analysis of sequenced genomes to identify bacteria that are capable of producing these structures has not been performed. To gain a comprehensive understanding of cellulosome displaying bacteria that could have applications in LCB degradation, we analyzed >305 k complete and draft microbial genomes for genes encoding cellulosomal proteins. This analysis revealed a total of 33 bacterial species have the capacity to produce cellulosomes, including 10 species not previously reported in the literature. These microbes produce simple or complex cellulosomes that are populated with either small or large numbers of DocGH enzymes that are known to degrade LCB. The majority of cellulosome-producing bacteria are members of the *Acetivibrio*, *Clostridium*, *Ruminiclostridium*, and *Ruminococcus* genera and exhibit phylogenetically conserved properties when their scaffoldins, enzymes, and mechanisms of cellulosomal gene regulation are compared (Figure 2).

## Results

### Identification of cellulosome displaying bacteria

**Figure 3:**
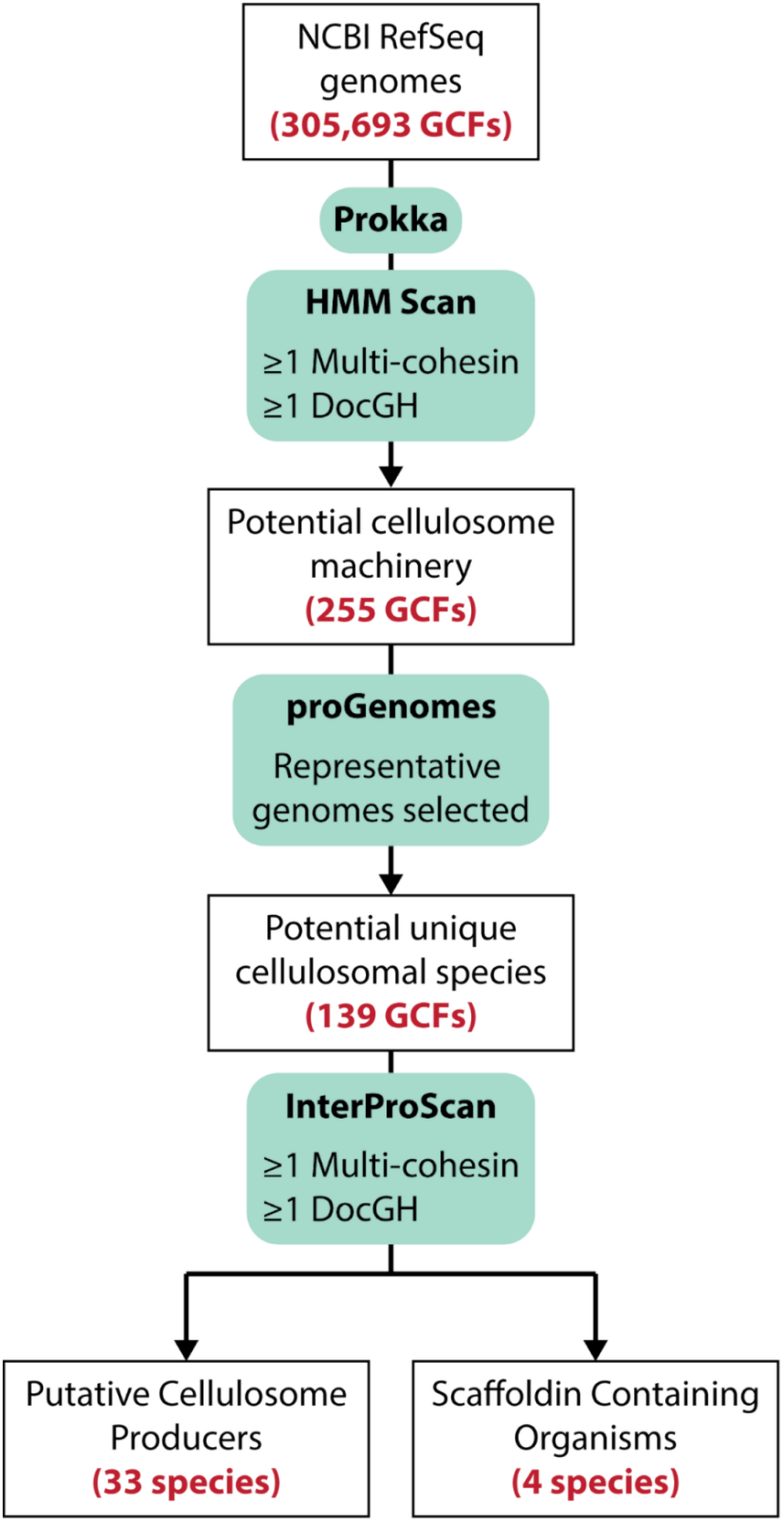
Strategy used to identify cellulosome producing microbes. More than 305 k sequenced bacterial genomes within the NCBI Reference Sequence database (RefSeq). Translated protein-encoding genes were then initially searched using HMM profiles to identify genomes that contained cohesin and DocGH enzymes. A subset of those genomes were then submitted for *de novo* annotation via Prokka (v. 1.14.16; Seemann, 2014) followed by a more stringent analysis of these protein sequences using InterProScan (v. 5.59-91.0; Quevillon et al., 2005) which identified 33 species that likely produce conventional cellulosomes based on the types of cohesin-containing proteins they possess. A total of 4 species contain multi-cohesin scaffoldins that are unlikely to assemble into conventional cellulosomes (Scaffoldin Containing Organisms).

To identify bacteria that display cellulosomes we searched a total of 305,693 prokaryotic genome assemblies within the NCBI Reference Sequence Database (RefSeq), which contains both complete and draft genomes (Figure 3; Li et al., 2021). Initially, the program HMMER (Eddy, 2009) was used to search the RefSeq-annotated protein-encoding genes for dockerin, cohesin, and GH domains using hidden Markov models (HMMs). A total of 255 genomes were carried forward for further analysis as they harbored at least one gene encoding a multi-cohesin containing scaffoldin (≥ 2 cohesin domains) and one gene encoding a DocGH protein. For cases where multiple genome sequences were available for the same bacterial species, only the representative genome within the ProGenomes database that contained the fewest number of contigs was analyzed (Mende et al., 2017). An exception was made for the three sequenced genomes from *A. thermocellus* (ATCC 27405, DSM 1313, and AD2), as it is a prototypical cellulosome-producing organism. After eliminating redundancies, a total of 139 distinct microbial genomes were retained for a more extensive and computationally demanding analysis using the program InterProScan (v. 5.59-91.0) (Quevillon et al., 2005). The analysis by InterProScan revealed a total of 37 bacterial species containing genes encoding putative cellulosomes (their genomes have at least one multi-cohesin and one DocGH encoding gene). Of these, 33 species likely produce conventional cellulosomes that are related to those in *C. acetobutylicum* and *A. cellulolyticus* (Figure 1), whereas 4 species may produce non-conventional cellulosomes (described below).

### Classification of bacteria based on their scaffoldin and DocGH enzyme content

**Figure 4:**
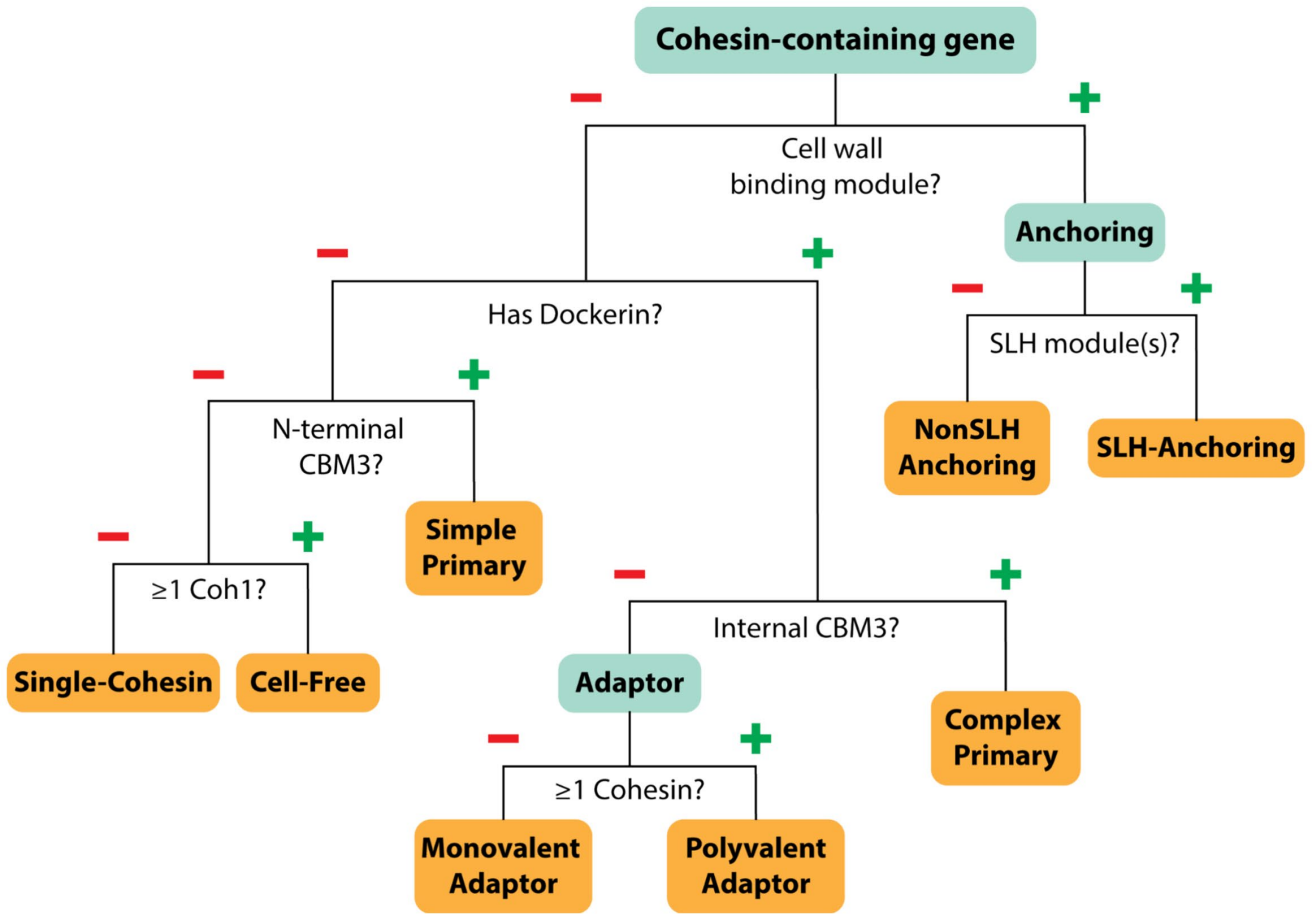
Scaffoldin categorization. Decision tree showing how each cohesin-containing protein was classified. Each cohesin-containing protein was classified into one of eight categories based on its type and abundance (Coh1, Coh2, Coh3), as well as the presence of additional domains such as dockerin, CBM3, and cell wall binding domains.

To gain insight into the structure and composition of each bacterium’s cellulosome, we systematically classified their scaffoldins into eight categories based on their domain content (Figure 4). The eight scaffoldin types and their functions in cellulosome assembly are demonstrated for *C. acetobutylicum* and *A. cellulolyticus*, which produce simple and complex cellulosomes, respectively (Figure 1; Dassa et al., 2012; Sabathé et al., 2002). These types include: (1) “simple primary” scaffoldins that are produced by *C. acetobutylicum* and other mesophiles which contain an N-terminal CBM3 and multiple cohesin domains (either Coh1 or Coh2) that are often interspersed with X2 domains (Artzi et al., 2017), (2) “single-cohesin” containing proteins of unknown function, (3) “complex primary” scaffoldins found in *A. cellulolyticus* and related species that harbor multiple Coh1 modules, an internal CBM3, and a single C-terminal Doc2 domain that enables it to bind to cell wall anchoring scaffoldins (Dassa et al., 2012), (4) “SLH-anchoring” scaffoldins that contain at least one cohesin module paired with a SLH-domain, (5) “nonSLH-anchoring” scaffoldins that contain at least one cohesin module and a known cell wall interacting domain/motif (e.g., LPxTG sorting signal, Lysin motif, C-terminal TM-helix, or Cu-Amine Oxidase-like domains), (6) “Monovalent adaptor” scaffoldins that contain a dockerin and a single cohesin module which have been proposed to facilitate type-switching between different types of cohesins and dockerin-fused enzymes (Artzi et al., 2017), (7) “Polyvalent adaptor” scaffoldins that contain a dockerin and several cohesins that suggest that more elaborate cellulosome architectures can be constructed by increasing the number of binding sites for DocGH enzyme proteins and/or scaffoldins (Artzi et al., 2017), and (8) “cell-free” scaffoldins that are presumably secreted to degrade LCB as they contain several cohesin domains that are capable of binding to DocGH enzymes (Raman et al., 2009). A list of the domain and motif identifiers used to discover cellulosome components within the cellulosome is provided in Supplementary Tables S1, S5.

**Figure 5:**
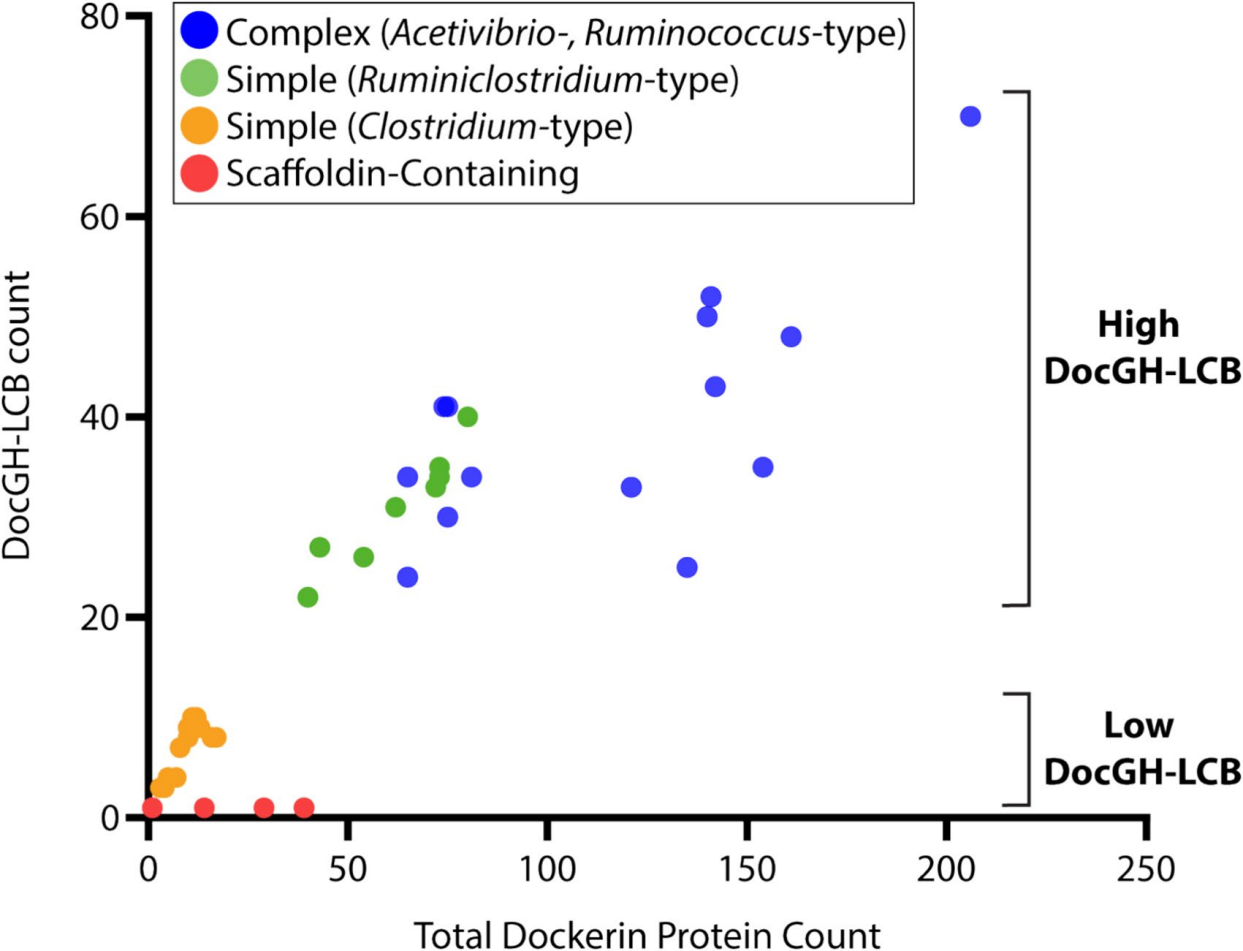
Dockerin-protein and DocGH-LCB distribution. Correlation between total dockerin protein count and DocGH-LCB protein count (*R*^2^ = 0.81, *p* < 0.05). Reported here are the number of genes encoding dockerin-fusion and DocGH-LCB proteins for all microbes listed in Tables 1, 2. Classes of microbes that are shown are indicated in the key and correspond to: Complex *Acetivibrio* and *Ruminococcus* cellulosomes (blue), Simple *Ruminiclostridium* cellulosomes (green), Simple *Clostridium* cellulosomes (orange), and bacteria containing scaffoldin encoding genes that are unlikely to produce conventional cellulosomes (red).

Next, we closely examined the enzyme composition of each bacterial species. The Carbohydrate-Active Enzyme database (CAZy) is a well-curated resource that classifies glycoside hydrolases (GHs), polysaccharide lyases (PLs), carbohydrate esterases (CEs), and glycosyl transferases (GTs) into families based on experimentally published data and publicly available sequences (Cantarel et al., 2009). GHs, PLs, and CEs are of particular interest in cellulosome-producing bacteria since their activities are directly linked to polymer breakdown. These enzymes are listed in Supplementary Table S1 and collectively referred to as CAZymes. Many of these CAZymes are often fused to a dockerin domain to facilitate their incorporation into cellulosomes, while enzymes not fused to dockerins are presumably secreted by the bacterium. Using the criteria outlined in Supplementary Table S1 we identified dockerin proteins fused GHs with activity against cellulose (DocGH-Cell), hemicellulose (DocGH-Hemi), and oligosaccharides (DocGH-Oligo), as well their corresponding non-dockerin-fused GHs (referred to as FreeGH-Cell, FreeGH-Hemi, and FreeGH-Oligo, respectively) (Supplementary Table S4). DocGH-Cell and DocGH-Hemi enzymes are of particular interest as they contribute significantly to cellulolytic activity against LCB and are distinguished as DocGH-LCB enzymes (DocGH-Cell plus DocGH-Hemi). A similar category was defined for non-dockerin-fused GHs with LCB activity (FreeGH-LCB). Based on their DocGH-LCB count there are two broad types of organisms that display cellulosomes, “high DocGH-LCB” microbes that contain genes encoding a large number of these enzymes (22 to 70) and “low DocGH-LCB” microbes that have fewer DocGH-LCBs (1 to 10) (Figure 5).

### Cellulosomes in *Ruminococcus* species

Our genomic screen detected all multi-cohesin cellulosome bacteria previously documented to produce cellulosomes, as well as several new species (Tables 1, 2). However, we detected fewer cohesin domain-containing proteins than previously reported in the literature for three genomes from two bacterial species, *R. champanellensis* (18P13) and *R. flavefaciens* (strains 17, 007c) (Ben David et al., 2015; Dassa et al., 2014). The genome for *R. flavefaciens* strain FD-1 previously described in the literature as containing a cellulosome was not analyzed by us because its sequenced genome is suppressed in the RefSeq database and because this species is already represented by several sequenced genomes (Dassa et al., 2014). For the remaining three genomes, there are two main reasons why their cohesin modules were undercounted. First, each genome contains a large number of contigs that lead to sequencing truncations in their cohesin-containing scaffoldin genes (Supplementary Table S2 lists the sequencing statistics for the genomes analyzed in this study). These truncations led to nine abbreviated cohesin-containing genes in *R. flavefaciens* strain 17, two in *R. flavefaciens* strain 007c, and four in *R. champanellensis*, lowering the number of detectable cohesins. For example, sequencing truncations in *R. flavefaciens* strain 17 occur in several of its scaffoldin genes (ScaB, ScaE, ScaG, ScaI, and orf02408) that previously were identified by sequencing a single contig from this microbe (Ding et al., 2001; Rincon et al., 2003; Rincón et al., 2004).

**Table 1:** Complex cellulosome producing bacteria.

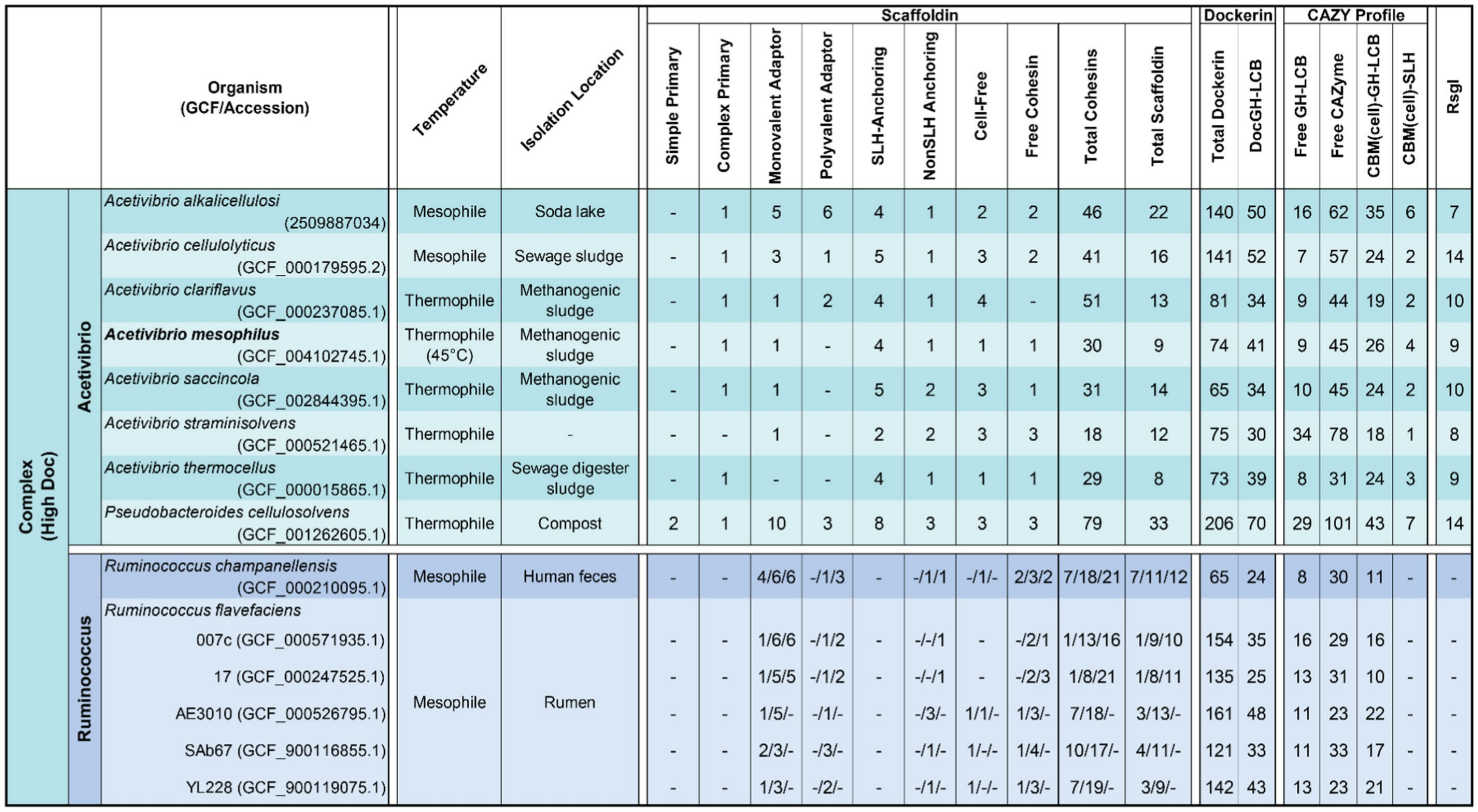

Tabulation of cellulosomal components for the complex cellulosome producing organisms identified in this study. The counts for the number of each scaffoldin type are reported (as defined in Figure 4). Also reported are the number per organism for the following: individual cohesin domains (Total Cohesin), scaffoldin proteins (Total Scaffoldin), all dockerin-containing proteins (Total Dockerin), LCB-active dockerin-fused GH enzymes (DocGH-LCB), non-dockerin-fused LCB-active GH enzymes (FreeGH-LCB), non-dockerin-fused GH, CL, and PE enzymes (including LCB active; Free CAZyme; Wang et al., 2022), LCB-active GH enzymes that are fused to a cellulose-binding CBM modules [CBM(cell)-GH-LCB], SLH-domains fused to a cellulose-binding CBM module [CBM(cell)-SLH], and transmembrane RsgI-like proteins (RsgI). A list of LCB active enzymes is provided in Supplementary Table S1. For the *Ruminococcus* species, three numbers are reported for each scaffoldin type. They are the values obtained when InterProScan (left) or AlphaFold2 (middle) was used to identify their cohesin modules and the scaffoldin number reported previously in the literature (right). Bolded organisms indicate those which have not been previously reported as capable of producing a cellulosome.

**Table 2:** Simple cellulosome producing bacteria.

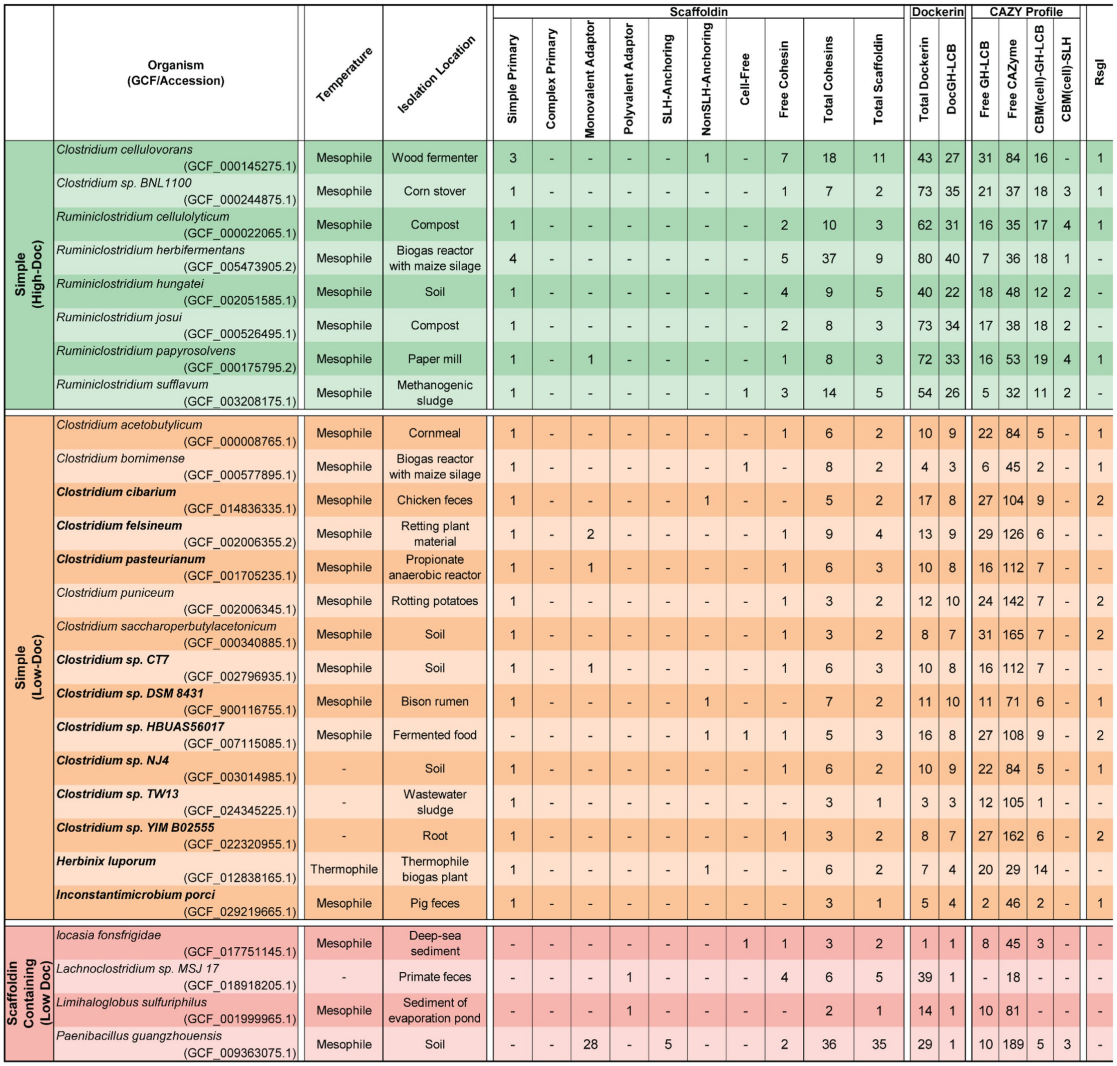

Tabulation of the components within the simple cellulosome producing bacteria that, respectively, contain either high (shaded green) or low numbers of genes encoding DocGH-LCB (shaded orange) enzymes. The table also shows data for four species that contain scaffoldins but are unlikely to produce conventional cellulosomes (shaded red). The counts for the number of each scaffoldin type are reported (as defined in Figure 1). The category definitions are identical to those presented in Table 1.

A second reason for the cohesin undercount is that the primary sequences of these modules in *Ruminococcus* species are highly divergent and thus not detectable using the HMM profiles employed by InterProScan (Coh1: cd08548, Coh2: cd08547, Coh3: cd08759, Coh: PF00963). For example, the protein encoded by the *scaA* gene in *R. flavefaciens* strain 007c has been reported to contain four cohesins and a C-terminal dockerin domain when its primary sequence was searched using BLAST and an unknown query sequence (Dassa et al., 2014). Instead, three of the modules are annotated as members of the CBM2/3 superfamily (SSF49384) and the fourth is not assigned to a protein family (Supplementary Table S3). Furthermore, in other *Ruminococcus* scaffoldins, previously reported cohesins are identified by InterProScan as G3DSA:2.60.40.680 superfamily members (CATH-3D) and not cohesins (Dawson et al., 2017). To investigate this issue we used AlphaFold2 to predict the atomic structures of scaffoldins within the *R. champanellensis* and *R. flavefaciens* genomes that contained cohesins that could not be identified by InterProScan. The predicted structures of these ‘undetected’ domains were compared to experimentally determined cohesin structures [Coh1 (PDB:1OHZ), Coh2 (PDB:2BM3) and Coh3 (PDB:2ZF9) cohesins] and their similarity determined by calculating a template modeling (TM) score (Dong et al., 2018). In all organisms, AlphaFold2-based predictions identified additional cohesin domains within scaffoldins that could not be detected by InterProScan (Supplementary Table S3). Given that we undercounted the cohesin domains in the three Ruminococcus species above, we extended the structural analysis to three additional *R. flavefaciens* strains (AE3010, SAb67, YL228) that were identified through our analysis. There is no literature reported cohesin domains for these strains. In *R. flavefaciens* strain AE3010, only three cohesin containing proteins (7 cohesin domains) were detected using InterProScan but based on AlphaFold2 a total of 18 domains (across 11 proteins) with cohesin folds are detected (summarized in Supplementary Table S3 and Supplementary Figure S2). Collectively, these results suggest that more complete genomic sequencing of *Ruminococcus* genomes is needed to fully define their scaffoldin complement and it highlights the utility of employing structure-based approaches to identify their cohesins.

## Discussion

Harnessing the potent cellulolytic activity of cellulosome producing microbes could lead to improved methods to convert abundant LCB into renewable chemicals and materials. To gain insight into their structures and distribution in nature, we searched 305,693 prokaryotic genomes for genes that encode cellulosome components—at least one multi-cohesin containing protein and one DocGH enzyme. This analysis identified 33 bacterial species that likely produce conventional cellulosomes that resemble those present in *C. acetobutylicum* and *A. cellulolyticus*, as well as 4 species that produce scaffoldin-containing structures that could bind DocGH enzymes and other dockerin-fusion proteins. The cellulosomes within the 33 species can be classified as having either complex or simple structures following the convention established by Bayer and colleagues (Figure 1; Tables 1 and 2) (Artzi et al., 2017). With only two exceptions, these microbes originate from four genera of gram-positive bacteria: *Acetivibrio, Ruminococcus*, *Ruminiclostridium*, and *Clostridium* (Figure 2). Based on their predicted complement of scaffoldin proteins, 10 species produce complex cellulosomes that are related to the one in *A. cellulolyticus,* while the remaining 23 species may produce less complex (simple) structures that resemble *C. acetobutylicum*’s cellulosome (Figure 1). We expect the cellulosomes in these bacteria to exhibit varying levels of cellulolytic activity based on their DocGH-LCB profiles (Figure 5; Supplementary Table S4). As documented in Table 1, all bacteria with the genetic capacity to produce complex cellulosomes also contain a large number of genes that encode DocGH-LCB proteins (called “high DocGH-LCB” microbes) and cellulose-binding CBM modules suggesting that they are cellulolytic. In contrast, the genomes of bacteria that display simple cellulosomes can either have high or low numbers of DocGH-LCB encoding genes, implying differences in their cellulolytic activities (Table 2). To the best of our knowledge, over a quarter of the cellulosome producing species discovered in this search (10 total) have not been previously described in the literature. Notably, although *R. cellobioparum* (*subsp. termitidis*; Dassa et al., 2017) and *R. bromii* (Ze et al., 2015) have previously been reported to produce cellulosomes, the sequenced genomes for these microbes lack genes for a multi-cohesin containing protein and therefore were not classified by us as containing a *bona fide* cellulosome. Below we summarize these findings.

### Complex cellulosome-producing bacteria

A number of bacterial species have been shown to display multi-scaffoldin containing complexes that have been referred to as “complex”/ “highly-structured” cellulosomes (Dassa et al., 2017; Artzi et al., 2017). Here we broadly define a complex cellulosome as containing at least two scaffoldins, a cell wall associated anchoring scaffoldin that contains a cohesin domain that could potentially bind to a dockerin domain located within a second multi-cohesin containing scaffoldin (Figure 1). Using this definition, complex cellulosomes contain either a complex primary scaffoldin (multi-cohesin, dockerin domain, and internal CBM3 domain containing) or a polyvalent adaptor (multi-cohesin and dockerin domain containing) that has the potential to bind via cohesin-dockerin interactions to the cell surface by interacting with an anchoring scaffoldin (either a SHL- or nonSLH-anchoring scaffoldin) (Figure 1). Based on this definition several *Acetivibrio* and *Ruminococcus* bacterial species produce complex cellulosomes (Table 1). *Acetivibrio* bacteria produce “classical” complex cellulosomes related the prototypical cellulosome from *A. thermocellus* in which DocGH enzymes bind to a complex primary scaffoldin that is tethered to the cell-surface via cohesin-dockerin interactions with a SLH-anchoring scaffoldin (Figure 1), whereas in *Ruminococcus* bacteria the enzymes bind to a polyvalent adaptor scaffoldin that form dockerin-cohesin interactions with a nonSLH-anchoring protein.

#### “Classical” complex cellulosomes in *Acetivibrio* species and *P. cellulosolvens*

Related complex cellulosomes are produced by 7 species of *Acetivibrio* bacteria [*A. alkalicellulosi* (Phitsuwan et al., 2019), *A. cellulolyticus* (Hamberg et al., 2014), *A. clariflavus* (Artzi et al., 2015), *A. mesophilus* (Rettenmaier et al., 2019), *A. saccincola* (Aikawa et al., 2018), *A. straminisolvens* (Kato et al., 2004), *A. thermocellus* (Lamed et al., 1983), and *P. cellulosolvens* (Zhivin et al., 2017)]. In the *Acetivibrio* bacteria, their complex primary scaffoldins contain multiple Coh1 domains that bind to DocGH enzymes through Doc1-Coh1 interactions and to the cell-surface through Doc2-Coh2 interactions with an SLH anchoring scaffoldin (Xu et al., 2004). *P. cellulosolvens* is the single exception, as it originates from the *Pseudobacteroides* genus and the roles of the cohesin-dockerin interactions are reversed (i.e., it uses Doc2/Coh2 and Doc1/Coh1 interactions to mediate DocGH and scaffoldin-scaffoldin binding, respectively) (Xu et al., 2004). In all of these bacteria the primary complex scaffoldin follow a similar domain arrangement, it contains multiple cohesins, a C-terminal dockerin module, and an internal CBM3 module that presumably enables each microbe to adhere to cellulose. These bacteria also possess Coh2-containing SLH-anchoring scaffoldins that bind to the bacterium’s peptidoglycan to coordinate the binding of the complex primary scaffoldin via its Doc2 domain. The lone exception is *A. straminisolvens*, which lacks a primary complex scaffoldin, but nevertheless contains a large number of other types of scaffoldins, including two SLH-anchoring scaffoldins. All of these bacteria are “high DocGH-LCB” producers and are characterized by the presence of a large number of accessory scaffoldins that increase the number of enzymes that can be incorporated into the cellulosome (Table 1). These accessory scaffoldins include monovalent adaptors, polyvalent adaptors, SLH anchoring, nonSLH anchoring, and cell-free scaffoldins (Figure 1). Both types of adaptor scaffoldins act to increase the size and complexity of the cellulosomes by extending the existing structure and allowing for type-switching within type-specific cohesin-dockerin interactions (Artzi et al., 2017). Aside from traditional SLH anchoring proteins, these bacteria have cohesin-containing nonSLH-anchoring scaffoldins that contain Cu-Amine Oxidase-like domains associated with secondary cell wall polymers (Dassa et al., 2012). The nonSLH-anchoring scaffoldins contain either Coh1 or Coh2 modules, suggesting they, respectively, facilitate either individual DocGH enzyme or complex primary scaffoldin binding to the cell surface. While not scaffoldins *per se*, *Acetivibrio* complex cellulosome producers also contain a large number of genes encoding CBM3-SLH fusion proteins that may function to tether the microbe to cellulose (Wang et al., 2022). The genomes of these microbes also contain a large number of genes encoding LCB active GHs that are fused to cellulose-binding CBM modules (Table 1, CBM(cell)-GH-LCB) as compared to other types of cellulosome producers. The cellulosomes in *Acetivibrio* bacteria are also unique because unlike other microbes, they uniformly produce multi-cohesin-containing cell-free scaffoldins that presumably form higher-order structures containing DocGH-LCB enzymes that are secreted into the environment to degrade LCB (Artzi et al., 2014; Chow and Wu, 2017).

*P. cellulosolvens* produces a complex cellulosome that is most closely related to those found in *Acetivibrio* species, as it contains SLH-anchoring scaffoldins that coordinate the binding of a complex primary scaffoldin bearing a C-terminal Doc1. Moreover, as compared to *Acetivibrio* and *Ruminococcus* bacteria, it is phylogenetically more closely related to *Acetivibrio* bacteria based on its 16S rRNA sequence (Figure 2). It stands out as producing the most complex cellulosome, since its genome encodes genes for an astounding 79 cohesin domains that are distributed between 33 scaffoldins: three primary, 10 monovalent adaptor, three polyvalent adaptor, eight SLH-anchoring, three nonSLH-anchoring, three cell-free scaffoldins, and three single cohesin domain containing proteins. *P. cellulosolvens* is also unique because it has genes encoding a complex primary scaffoldin and two primary scaffoldins that are typically found in simple cellulosomes within *Ruminiclostridium* and *Clostridium* bacteria (previously referred to as ScaM1 and ScaM2 in *P. cellulosolvens*; Zhivin et al., 2017). As noted previously, the usage of the cohesin and dockerin domains in *P. cellulosolvens* is reversed as compared to other bacteria in the *Acetivibrio* category.

Another conserved feature in *Acetivibrio* bacteria and *P. cellulosolvens* is the manner in which they control the expression of the DocGH enzymes and scaffoldin proteins to construct their cellulosome. Microbes have been shown to alter the complement of their DocGH enzymes when different types of LCB substrates are encountered (Artzi et al., 2015; Blouzard et al., 2010) by regulating gene expression using either two-component systems (Celik et al., 2013; Kampik et al., 2020), selective RNA transcript stabilization (Bhaskar et al., 2021; Xu et al., 2015), or transmembrane biomass-sensing RsgI-type anti-σ factors that regulate σ^I^-factors (Nataf et al., 2010; Muñoz-Gutiérrez et al., 2016; Kahel-Raifer et al., 2010). Our analysis suggests that in *P. cellulosolvens* and all *Acetivibrio* complex cellulosome producing bacteria, RsgI-type anti-σ factors are used to dictate cellulosomal gene expression, as each species contains genes encoding 7 to 14 of these factors (Table 1).

#### Complex cellulosomes in *Ruminococcus* species

Previous studies have shown that *Ruminococcus flavefaciens* and *Ruminococcus champanellensis* produce cellulosomes, which like their *Acetivibrio* counterparts contain an array of primary, cell wall anchoring, and adaptor scaffoldins (Artzi et al., 2017). Prior studies highlighted several unique features in *Ruminococcus* cellulosomes. First, cell surface attachment in *Ruminococcus* cellulosomes is often mediated by an anchoring scaffoldin that is covalently linked to the cell wall by a sortase enzyme instead of by a SLH-anchoring scaffoldin as observed in *Acetivibrio* species (Dassa et al., 2014; Rincon et al., 2007). Second, the primary scaffoldins in *Ruminococcus* species lacks a characteristic internal CBM3 that can mediate direct attachment to cellulosic substrates. Third, unlike *Acetivibrio* bacteria, these species contain a unique monovalent adaptor scaffoldin called ScaC which is often used as the genomic signature to identify *Ruminococcus* cellulosomes (Rincón et al., 2004; Jindou et al., 2008). Lastly, it has been noted in the literature that the scaffoldins in the *Ruminococcus* cellulosomes contain dockerin and cohesin modules with divergent primary sequences (frequently Doc3-and Coh3-types; Ding et al., 2001; Salama-Alber et al., 2012).

The scaffoldin proteins were identified in *R. champanellensis* 18P13 and *R. flavefaciens* (strains 17 and 007c) before their genomes were sequenced (Dassa et al., 2014). Surprisingly, we identified fewer cohesin domains and scaffoldin proteins encoded in these genomes than previously reported. In many cases this occurred because their sequenced genomes contain a large number of sequence contigs that caused scaffoldin gene truncations (Supplementary Table S2). In other instances, even if an intact, non-truncated scaffoldin encoding gene was present, it was not possible to detect the full complement of cohesins within the translated protein product using the sequence profiles employed by InterProScan (Coh1: cd08548, Coh2: cd08547, Coh3: cd08759, Coh: PF00963). Indeed, only when AlphaFold2 was used to predict the atomic structures of these proteins was the full complement of previously reported cohesin modules identified. For example, an InterProScan analysis of translated genes in *R. flavefaciens* (strain 007c) identified a single scaffoldin, whereas 10 scaffoldins have been reported in the literature (Dassa et al., 2014). However, when AlphaFold2 was employed, 9 of these 10 scaffoldins were detected. Similar results were obtained when AlphaFold2 was applied to other ruminococcal genomes documented to contain genes for cellulosomes (summarized in Supplementary Table S3). Interestingly, even though Coh3 domains are a signature feature of ruminococcal cellulosomes, InterProScan did not identify these domains in *R. champanellensis* 18P13 or *R. flavefaciens* (using the cd14255 profile for a Coh3 domain). We conclude that the sequence signatures employed by InterProScan are not sufficiently robust to identify Coh3 cohesins, consistent with the findings reported by Flint and colleagues who have subdivided *R. flavefaciens’* cohesins into 6 different groups based on sequence homology (Ding et al., 2001). Our results also demonstrate the utility of using AlphaFold2 structure predictions to identify cohesins with divergent primary sequences.

#### *Acetivibrio mesophilus* may produce a “classical” complex cellulosome

**Figure 6:**
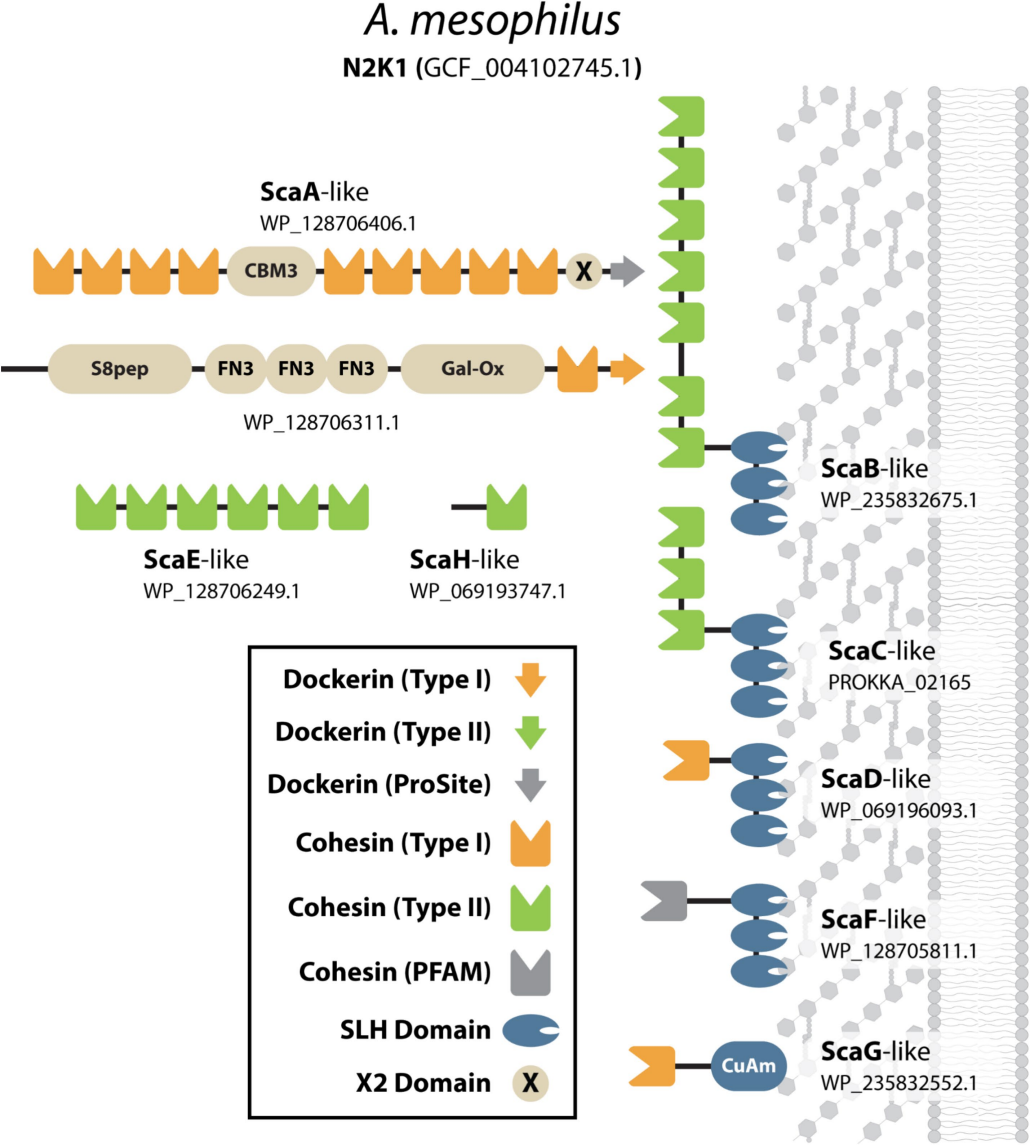
Scaffoldin proteins in *A. mesophilus* (N2K1). Cartoon representation of all of the cohesin-containing proteins in *A. mesophilus* (N2K1). The domains they possess are defined in the key and also include; Fibronectin Type-III like (FN3), CBM type-3 domain (CBM3), Copper Amine Oxidase-Like (CuAm), S8-subtilase/peptidase (S8pep), and Galactose Oxidase-like (Gal-Ox) domains. Proteins were named based on their sequence homology to scaffoldins present in *A. thermocellus*.

Our analysis identified a previously unrecognized bacterium as a “classical” complex cellulosome producer, *Acetivibrio mesophilus* N2K1 (formerly known as *Hungateiclostridium mesophilum*) (Tindall, 2019). This gram-positive anaerobic bacterium was first isolated from a mesophile consortium in a biogas fermenter fed with maize silage (Rettenmaier et al., 2019). It has an optimal growth temperature of 45°C and expresses two hemicellulases that have been biochemically characterized, but the presence of a cellulosome has not been reported to the best of our knowledge (Liu et al., 2021). Its cellulosome is likely cellulolytic because its genome contains 40 genes encoding DocGH-LCBs. Based on our analysis, *A. mesophilus*’ cellulosome is strikingly similar to the archetypal cellulosome produced by *A. thermocellus* (Figure 6). Specifically, both species produce a “classical” complex primary scaffoldin (ScaA-like: WP_128706406.1) that contains nine Coh1 modules, an internal CBM3, and a C-terminal Doc2 module for cell surface attachment via interactions with a SLH-anchoring scaffoldin (ScaF-like: WP_128705811.1). *A. mesophilus* has an additional three genes for SLH-anchoring scaffoldins (ScaB-like: WP_235832675.1, ScaC-like: PROKKA_02165, and ScaD-like: WP_069196093.1) that are related to *A. thermocellus*’ ScaB, ScaC, and ScaD scaffoldins, as well as one nonSLH-anchoring scaffoldin (ScaG-like: WP_235832552.1) that is similar to ScaG. *A. mesophilus* also produces an additional scaffoldin that closely resembles scaffoldins found in *C. alkalicellulosi* (ScaO2; Phitsuwan et al., 2019) and *A. cellulolyticus* (ScaO; Dassa et al., 2012). This scaffoldin (WP_128706311.1) contains a C-terminal Coh1-Doc1 bi-domain unit, three Fibronectin-type III (FN3) repeats, as well as S8-peptidase-like and galactose oxidase-like domains of unknown function. Given the presence of a C-terminal Doc1 domain in this scaffoldin, it is tempting to speculate that it binds to the primary scaffoldin and/or the ScaD-like and ScaG-like scaffoldins that contain complementary Coh1 modules.

### Bacteria that produce simple cellulosomes

We identified 23 species of anaerobic mesophilic bacteria that display less complex cellulosomes and fewer types of scaffoldins. The cellulosomes in these microbes always contain a simple primary scaffoldin that houses several Coh1 domains and an N-terminal CBM3 module (Figure 1). In all cases the gene encoding the primary scaffoldin protein (*cipA*) is located within a *cipA* operon that also contains genes for DocGH enzymes. The vast majority of DocGHs in these microbes contain complementary Doc1 modules for direct interaction with the simple primary scaffoldin. The lone exception is *Clostridium* sp. *HBUAS56017*, whose simple primary scaffoldin lacks a CBM.

Simple cellulosome producing bacteria originate from the *Ruminiclostridium* and *Clostridium* genera (Figure 2). The *Ruminiclostridium* bacteria produce simple cellulosomes that likely have high cellulolytic activity as their genomes contain a large number of DocGH-LCB genes [*R. hungatei* (Monserrate et al., 2001), *R. papyrosolvens* (Ren et al., 2019), *R. josui* (Kakiuchi et al., 1998), *R. herbifermentans* (Rettenmaier et al., 2019), *R. cellulolyticum* (Desvaux, 2005), and *R. sufflavum* (Nishiyama et al., 2009) (Figure 5)]. In contrast, nearly all of the clostridial species are low DocGH-LCB producers suggesting their simple cellulosomes have limited cellulolytic activity [*C. felsineum* (previously known as *C. roseum*; Collins et al., 1994), *C. acetobutylicum* (Sabathé et al., 2002; López-Contreras et al., 2003; López-Contreras et al., 2004), *C. bornimense* (Tomazetto et al., 2016), *C. cibarium* (Gilroy et al., 2021), *C. puniceum* (Dassa et al., 2017), *C. saccharoperbutylacetonicum* (Levi Hevroni et al., 2020), and several additional *Clostridium* spp. (CT7, HBUAS56017, DSM 8431, NJ4, TW13, YIM B02555)]. This notion is consistent with experimental data, as the simple cellulosomes produced by high number DocGH-LCB bacteria, *R. cellulolyticum, R. papyrosolvens,* and *R. herbifermentans,* are shown to have potent cellulolytic activity (Ren et al., 2019; Rettenmaier et al., 2019; You et al., 2023; Giallo et al., 1985), whereas the simple cellulosomes in *C. acetobutylicum* and *C. saccharoperbutylacetonicum* that have a low number of DocGH-LCB genes are less cellulolytic (Sabathé et al., 2002; López-Contreras et al., 2003; López-Contreras et al., 2004; Levi Hevroni et al., 2020). The only two exceptions to the idea that simple cellulosomes in clostridial bacteria are less cellulolytic are from *C. cellulovorans* (Usai et al., 2020) and *Clostridium* sp. BNL1100 (Li et al., 2012). These species are presumably cellulolytic as their genomes contain a high number of DocGH-LCB encoding genes. However, it is noteworthy that based on its 16S rRNA sequence, *Clostridium* sp. BNL1100 can be classified as a member of the *Ruminiclostridium* genus (Figure 2). Finally, there are two “non-clostridial” species with genomes encoding simple cellulosomes and a low number of DocGH-LCB enzymes, *Herbinix luporum* (Koeck et al., 2016) and *Inconstantimicrobium porci* (Wylensek et al., 2020). Based on their 16S rRNA sequence both species are most closely related to clostridial bacteria.

The mechanism(s) through which simple cellulosomes are attached to the cell surface remains incompletely understood. This is because their primary simple scaffoldins lack obvious cell wall binding modules or dockerin domains that could mediate their attachment to anchoring scaffoldins (either SLH-anchoring or nonSLH-anchoring scaffoldins; Figure 1). Cell wall attachment by simple primary scaffoldins may be mediated by their N-terminal CBM3 domain as supported by recent Western blot data demonstrating cell surface binding by a CBM3a module (Tao et al., 2022). In addition, all of the simple primary scaffoldins contain X2 domains that have been implicated in both cell wall attachment (Kosugi et al., 2004) and cellular interactions with cellulose (Tao et al., 2022; Tarraran et al., 2021). Notably, two bacterial species contain genes for more than one primary simple scaffoldin, *R. herbifermentans* and *C. cellulovorans* (Table 2). *R. herbifermentans’* genome has genes encoding four simple primary scaffoldins that are located in a single *cipA* operon (Rettenmaier et al., 2019), with each scaffoldin possessing 5 to 14 Coh1 modules. In the case of *C. cellulovorans*, genes for 3 primary scaffoldin proteins are present (WP_010073402.1, WP_010073403.1, and WP_013291799.1). Of these, only one scaffoldin (WP_013291799.1, CbpA) is encoded by a gene located within a *cipA* gene cluster (Dassa et al., 2017).

Some species of simple cellulosome-producing bacteria harbor genes encoding unique accessory scaffoldins with large numbers of FN3 domains that have been proposed to disrupt crystalline polysaccharide structures or solubilize large protein complexes (Kataeva et al., 2002; Alahuhta et al., 2010). Simple cellulosome displaying bacteria containing FN3 scaffoldins include: *R. cellulolyticum, R. herbifermentans, R. hungatei, C. josui, R. sufflavum, C. felsineum, C. pasteurianum*, and *Clostridium* sp. CT7. *C. pasteurianum* and *Clostridium* sp. CT7 are notable as they each contain a scaffoldin with 30 FN3 repeats that is capped by a C-terminal Coh2-Doc2 module whose binding partners are unknown, as no other proteins in these microbes contain Doc2 or Coh2 modules. A limited number of complex cellulosome-displaying bacteria also contain scaffoldins with FN3 domains, but typically only 1–3 copies of this module are present. Finally, three simple cellulosome-producing bacteria produce cell-free scaffoldins that may be secreted as they lack dockerin domains and cell wall binding modules (*R. sufflavum*, *Clostridium* sp. HBUAS56017, and *C. bornimense*).

#### Novel and previously uncharacterized simple cellulosome displaying bacteria

We identified 9 new bacterial species that based on their genome sequences produce simple cellulosomes: *Clostridium cibarium*, *Clostridium pasteurianum, Clostridium* sp. CT7, *Clostridium* sp. DSM 8431, *Clostridium* sp. HBUAS56017, *Clostridium* sp. NJ4, *Clostridium* sp. TW13, *Clostridium* sp. YIM B02555, and *I. porci*. Supplementary Figure S1 shows their predicted scaffoldins, while Supplementary Table S4 enumerates their dockerin, cohesin, and enzyme content. They are all low DocGH producers that phylogenetically cluster with clostridial bacteria (Figure 2). Each contains at least one simple primary scaffoldin that is a hallmark of simple cellulosome producers—defined as a scaffoldin that contains two or more cohesin domains and an N-terminal CBM3 (a lone exception is *Clostridium* sp. CT7 that lacks an N-terminal CBM3). As with other simple cellulosome producers, their primary scaffoldins contain X2 and Coh1 modules and each microbe almost exclusively produces DocGH enzymes containing Doc1 modules. Several of these microorganisms also produce a single scaffoldin that encodes 1–2 cohesins which are either Coh1-or Coh2-type (*Clostridium* sp. NJ4*, Clostridium* sp. YIM B02555*, Clostridium* sp. HBUAS56017*, Clostridium* sp. DSM 8431*, Clostridium* sp. CT7, *Clostridium cibarium*).

Our analysis predicts for the first time the scaffoldin and enzyme composition in 4 microbes previously noted to produce cellulosomes: *R. hungatei*, *C. bornimense*, *C. felsineum*, and *H. luporum* (Supplementary Table S4). All have the genomic capacity to produce simple primary scaffoldins that are the core of a simple cellulosome (Figure 1). *R. hungatei* DSM 14427 is of particular interest as it is the only one in this group whose genome contains a high number of DocGH-LCB encoding genes, as well as genes encoding 3 accessory scaffoldins that contain 7–9 FN3 modules and a C-terminal cohesin domain. Based on their primary sequences, 2 of these scaffoldins contain Coh2 domains whose binding partners are not known because *R. hungatei*’s genome lacks genes that encode for Doc2 containing proteins. Finally, the genomes of *H. luporum* and *C. cibarium* encode for proteins that may function as nonSLH-anchoring scaffoldins, as they contain a single Coh2 domain and a C-terminal transmembrane helix that may be embedded in the bilayer. However, the binding partners for these scaffoldins also remain unclear, since only in *C. cibarium* are genes encoding complementary Doc2 containing proteins identifiable.

### “Scaffoldin-containing” microbes

We used broad search criteria to identify cellulosome producing bacteria—any genome that contained a gene for at least one multi-cohesin and one DocGH protein. Four microbial genomes barely satisfied these criteria and are unlikely to produce conventional cellulosomes because their largest scaffoldin contains only two cohesin modules. These include three species from the gram-positive *Bacillota* phylum whose members are known to display cellulosomes, *Iocasia fonsfrigidae* (Zhang et al., 2021), *Lachnoclostridium* sp. MSJ-17, and *Paenibacillus guangzhouensis* (Li et al., 2014), as well as *Limihaloglobus sulfuriphilus* (Pradel et al., 2020) which is a member of the rare Planctomycetes–Verrucomicrobia–Chlamydiae (PVC) superphylum. *Lachnoclostridium* sp. MSJ-17 encodes four cohesin-containing scaffoldins, three proteins that contain a single cohesin, and one larger scaffoldin that contains two cohesins and an N-terminal Doc1 domains. Two of the single cohesin-containing scaffoldins may be cell-associated as they contain C-terminal transmembrane helices (WP_216523161.1 and WP_216522914.1). Collectively, these scaffoldins could bind as many as 39 distinct dockerin-containing proteins, but the microbe is presumably non-cellulolytic as its genome encodes only a single DocGH-LCB. *I. fonsfrigidae* was isolated from deep sea sediment and has the potential to produce two large scaffoldins. One of them contains two cohesins, an FN3 module, and a CBM3 domain that could mediate cellulose binding. The second scaffoldin is also sizable (662 amino acids) but is predicted to contain only a single C-terminal cohesin domain. Interestingly, this microbe’s genome contains only a single gene encoding one DocGH-LCB enzyme. The gram-positive soil bacterium *P. guangzhouensis* is the most impressive of the scaffoldin-containing microbes as its genome encodes 35 cohesin-containing proteins. A total of 28 of these proteins are monovalent adaptors that contain a single dockerin-cohesin domain pair and in many instances, they possess an additional N-terminal GH enzyme that could degrade hemicellulose or oligosaccharide polymers (GH2, GH20, GH29, GH31, and GH43). There are also five SLH-anchoring scaffoldins to which these proteins could dock onto, potentially creating an enzyme rich surface that would be architecturally distinct from conventional cellulosomes (Phitsuwan et al., 2019). Fascinatingly, in this microbe, nearly all of its dockerin domains are located within its cohesin-containing scaffoldins (only one dockerin is located in a non-scaffoldin protein). This raises the possibility that the full-complement of its dockerin proteins were not detected by InterProScan and/or its scaffoldins act to opportunistically scavenge dockerin-fusion proteins that are produced by other microbes. Notably, *P. guangzhouensis’* genome also contains a large number of genes that encode for CAZymes, but most of these are not of the family-type that is known to degrade LCB. The genome in the PVC superphylum bacterium *L. sulfuriphilus* contains genes encoding a single Coh2-Doc1-Coh1 scaffoldin-like protein and 13 dockerin-fusion proteins. It is presumably non-cellulolytic as it contains only a single DocGH-LCB and a limited number of CAZymes. Notably, the dockerin proteins in *L. sulfuriphilus* are fused to domains not commonly found in cellulosome producing bacteria, including FAD/NAD-binding, aspartic peptidase-like domains, and HdrA-like domains.

### Phylogenetic variation in dockerins and CAZymes

**Figure 7:**
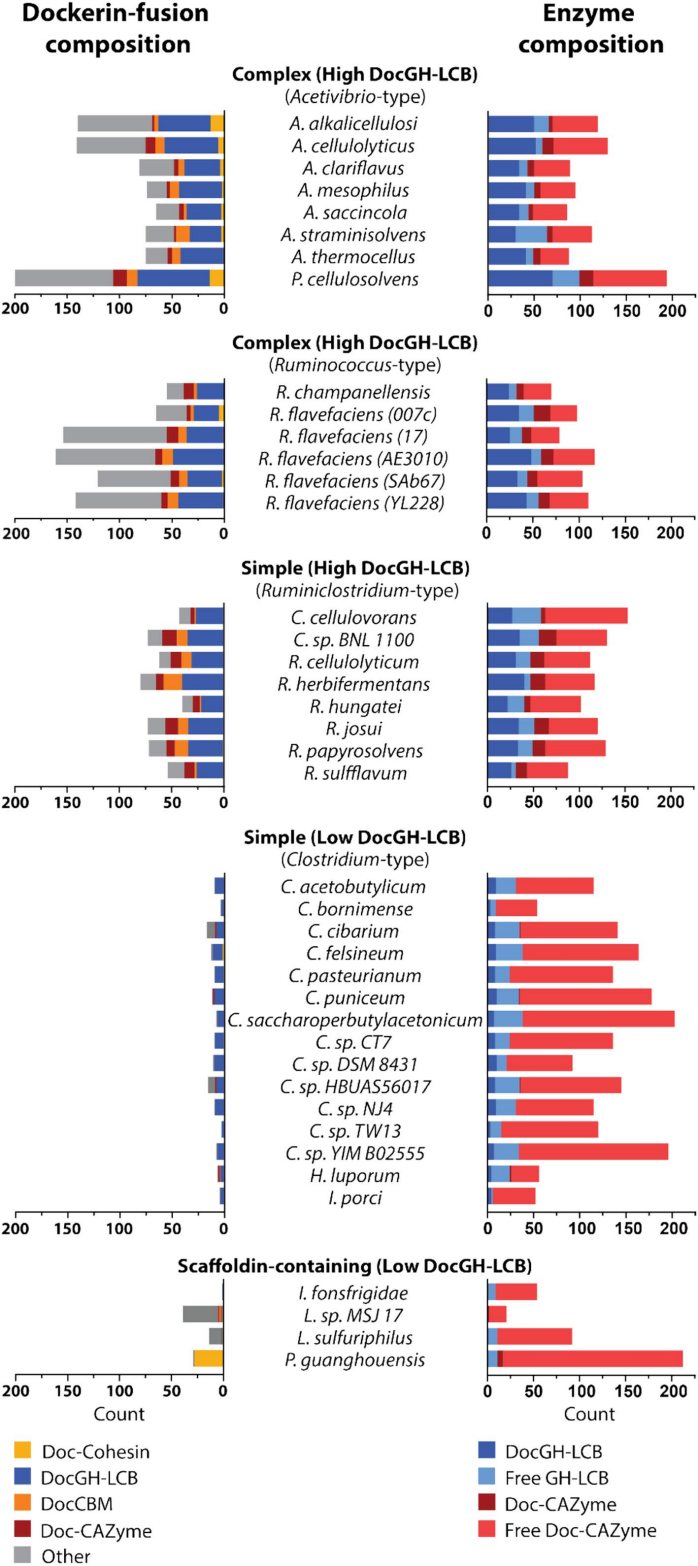
Domain and enzyme composition in dockerin-fused proteins. Plots show the types of domains that are fused to dockerin proteins in microbes that produce *Acetivirbio*-type complex cellulosomes, *Ruminococcus*-type complex cellulosomes, *Ruminiclostridium*-type simple cellulosomes, *Clostridium*-type simple cellulosomes, and other bacteria with scaffoldin-containing proteins that are unlikely to form conventional cellulosomes (see Tables 1, 2 for a complete list). (Left) Stacked bar plot representation of the number of dockerin-fusion proteins based on the types of domains they contain. Co-occurring domain type is color coded according to the following: Yellow: Cohesin, Dark Blue: Glycoside Hydrolase with LCB activity, Orange: Carbohydrate Binding Module, Red: Carbohydrate Active Enzymes (CAZyme), Grey: other. (Right) Stacked bar plot representation of cellulosomal enzyme composition for each cellulosome-producing microbe, including; Dark Blue: DocGH-LCB, Light Blue: Free GH-LCB, Red: Dockerin-fused CAZyme, and Light Red: Free CAZyme.

Across the studied bacterial species in this study, an examination of their dockerin-fusion proteins provides insight into both the numbers and types of proteins that are incorporated into cellulosomes (Figures 5, 7). In general, the genomes of *Acetivibrio* and *Ruminococcus* bacteria produce complex cellulosomes containing a high number of DocGH-LCB genes (>20) suggesting that these structures are cellulolytic (Figure 5). In contrast, *Ruminiclostridium* and *Clostridium* bacteria that have the genomic capacity to produce simple cellulosomes contain either high or low numbers of DocGH-LCB genes, respectively. Interestingly, a near linear relationship is observed between the total number of dockerin and DocGH-LCB encoding genes within an organism (R^2^ = 0.81), which is consistent with the primary function of cellulosomes being to degrade LCB (Figure 5). Thus, only bacteria whose genomes contain a large and diverse set of DocGH-LCBs (*Ruminiclostridium*, *Acetivibrio*, and *Ruminococcus*) also possess a high number of genes that encode for other types of dockerin-fusion proteins. For example, on average high DocGH-LCB producers contain ~40 genes encoding DocGH-LCBs and an impressive ~80 genes that encode other types of dockerin-fusion proteins (Figure 7). This number is much smaller in the low DocGH-LCB producers (*Clostridium*), as they only contain on average ~ 2 and ~ 3 genes that encode DocGH-LCBs and other types of dockerin-fusions, respectively. There is significant variability amongst the high DocGH-LCB producing microbes, as *P. cellulosolvens* (classified by us as an *Acetivibrio*-type) contains a total of 206 dockerin-fusion genes (of which 70 are DocGH-LCB genes), whereas *R. hungatei* (*Ruminiclostridium*-type) contains only 40 (20 DocGH-LCB genes). Interestingly, our analysis reveals that bacteria frequently supplement their DocGH-LCBs with a similar set of dockerin-fused accessory proteins that may facilitate LCB degradation. These include dockerin-fusion proteins containing: CBMs that bind carbohydrates (Doc-CBM, orange), carbohydrate active hydrolases such as pectin lyases and carbohydrate esterases (Doc-CAZymes, red), cohesin domains that are part of scaffoldins that construct the cellulosome (Doc-cohesin, yellow), and proteins with other functions (Doc-other, gray; Figure 7). This enrichment supports the idea that the primary function of the cellulosomes in *Acetivibrio, Ruminococcus*, and *Ruminiclostridium* bacteria is to degrade LCB or related carbohydrate polymers. Interestingly, perhaps to compensate for their deficiency in DocGH-LCBs and complementary proteins, the genomes of some clostridial bacteria contain larger numbers of genes encoding carbohydrate active hydrolases that are freely secreted (Figure 7).

## Conclusion

Our results indicate that with only a few exceptions, bacteria with the genetic capacity to produce cellulosomes originate from four genera: *Acetivibrio*-*, Ruminococcus*-, *Ruminiclostridium-* and *Clostridium*-type cellulosomes. *Acetivibrio*-type cellulosomes, including the one found in *P. cellulosolvens*, are complex and can be populated with a high number distinct LCB active enzymes. They are characterized by the presence of a conserved dockerin-containing primary scaffoldin, SLH-anchoring scaffoldins that tether the cellulosome to the cell surface, cell-free scaffoldins that are presumably secreted to form multi-enzyme complexes, and CBM3-SLH proteins that may enable microbial tethering to LCB. Dockerin-fused enzymes bind to the scaffoldins via Doc1-Coh1 interactions, whereas Doc2-Coh2 interactions mediate scaffoldin-scaffoldin interactions (the exception is *P. cellulosolvens* in which these interactions are reversed). Only microbes that harbor *Acetivibrio*-type cellulosomes use a dedicated suite of polysaccharide-sensing RsgI transmembrane receptors to regulate its composition. *Ruminococcus* bacteria (*R. champanellensis* or *R. flavefaciens*) can also produce complex cellulosomes that contain a high number of distinct DocGH-LCB enzymes. However, they are unique because their anchoring scaffoldins are attached to the cell wall via sortase enzymes instead of SLH domains, and many of their dockerin-fusion proteins contain Doc3 modules. It was challenging to define the components of these structures from genomic sequence data as their cohesins frequently have divergent primary sequences that could not be detected using InterProScan. Indeed, we detected no Coh3-type modules based on their primary sequence, and only when AlphaFold2 was employed to predict their structures were several cohesins identified. *Ruminiclostridium* and *Clostridium* bacteria produce ‘simple’ cellulosomes that contain only a limited number of scaffoldins. They are further distinguished by the presence of a primary scaffoldin that contains an N-terminal CBM3, X2, and multiple cohesin domains. Their genomes encode only a limited number of scaffoldins and their primary scaffoldins adhere to the microbial surface through a poorly understand mechanism as they lack obvious domains that are capable of binding to the cell wall. These simple cellulosomes can be subdivided further by the number of distinct DocGH-LCB enzymes they house, with *Ruminiclostridium* and *Clostridium* genomes typically encoding for high and low numbers of DocGH-LCB enzymes, respectively. Finally, several species of simple cellulosome displaying bacteria are unique as they contain scaffoldins harboring up to 30 FN3 repeats that may disrupt crystalline polysaccharide structures and/or solubilize large protein complexes. Mapping the precise architectures of these cellulosomes requires additional experimental studies to define the specific set of cohesin-dockerin interactions that form the “glue” that hold these structures together, since at present it is not always possible to reliably predict the specificity of these interactions using only primary sequence data.

This comparative genomic analysis identified 33 bacterial species with the capacity to produce cellulosomes, including 10 previously unreported species. The actual number of distinct cellulosome producing species in nature is likely much larger, as up to 1.6 million operational taxonomic units (a proxy for bacterial species) are estimated to exist (Louca et al., 2019), of which only ~2.1% have had their genomes completely sequenced (Zhang et al., 2020). This undersampling is evident from metagenomics data, which reveals the presence of cellulosome displaying bacteria with incompletely sequenced genomes. Here we focused our efforts only on bacteria with completely sequenced genomes, as significant genome gaps exist in metagenomic data that make it extremely difficult to identify the complete set of cellulosome encoding genes in these microbes (Nam et al., 2023). Collectively, the results of this study provide insight into the structural diversity of bacterial cellulosomes, and they reveal conserved architectural features that may be useful in guiding ongoing engineering efforts to produce bio-based chemicals and materials from plant biomass.

## Materials and methods

### Genome-based search to identify cellulosome displaying bacteria

The retrieval of cellulosome-displaying bacteria consists of two components: (i) a pre-scan phase to select for genomes that potentially contain cellulosomes, and (ii) an in-depth scan of each of these filtered genomes. While metagenomic sequencing has strengths in identifying novel microbes, the aim of this study is to categorize and take inventory of the diversity of cellulosomes displayed by fully sequenced microbes. For this analysis we therefore did not include metagenome-based sequences because genomes resolved by this method are often incomplete and constructed from many contigs (Nam et al., 2023). These two shortcomings result in significant gaps in an organism’s genome making it difficult to resolve all protein coding genes. Therefore, for the pre-scan phase, only the RefSeq database (O'Leary et al., 2016; July 10, 2023 release) was used, which contained 138,491 organisms with 371,291,248 records. The genome of *Acetivibrio alkalicellulosi* is not deposited in the RefSeq database, because it has been suppressed due to contamination (Supplementary Table S2). However, it has been reported to produce a cellulosome and therefore was manually included in our analysis (Phitsuwan et al., 2019). From the RefSeq database we retrieved only prokaryotic genomes (305,693) and performed a hidden Markov model (HMM) search (Potter et al., 2018) using dockerin (cd14256, cd14254, cd14255), cohesin (cd08548, cd08547, cd08759), and GHs (PF00150, PF03537, PF01670, PF12891, PF02015, PF02011, PF01270, PF00759) domain profiles obtained from the Conserved Domains Database (CDD) and Pfam database (Wang et al., 2023; Mistry et al., 2021). Genomes were targeted for the next in-depth phase only if they met the following criteria: i) they must have at least one dockerin-fused GH protein, and ii) they must have at least one protein with two or more cohesins.

In the in-depth scanning phase, the 139 genomes from the prescan phase were re-annotated using Prokka (v. 1.14.6; Seemann, 2014), and all the locus tags from NCBI’s annotations were mapped using BLAST (v. 2.13.0+; Camacho et al., 2009) to maintain consistency with the existing gene naming convention. After re-annotation, InterProScan (v. 5.59-91.0; Jones et al., 2014) was performed, which includes the following member databases: Phobius (v. 1.01; Käll et al., 2004), SUPERFAMILY (v. 1.75; Gough et al., 2001), ProSiteProfiles (v. 2022_01; Sigrist et al., 2013), SMART (v. 7.1; Letunic et al., 2021), CDD (v. 3.18; Wang et al., 2023), PRINTS (v. 42.0; Attwood et al., 2012), and Pfam (v. 35.0; Mistry et al., 2021). Additionally, three databases were added to capture signal peptide regions (SignalP v. 6.0; Teufel et al., 2022), transmembrane regions (DeepTMHMM v. 1.0.19; Hallgren et al., 2022), and subcellular localization information (PSORTb v. 3.0; Yu et al., 2010).

After the in-depth genome analysis, we applied the same criteria as before (i) ≥1 DocGH fusion protein and (ii) at least one protein with ≥2 cohesin domains) and identified 37 genomes that met our criteria. 132 genomes did not pass our criteria using the InterProScan data because the initial HMMER search was not conducted with stringent threshold values, which inflated the initial number of domains each genome had. However, this genome-based analysis procedure did not identify several bacteria which have previously been shown to display cellulosomes (e.g., *Ruminococcus flavefaciens* 007c, FD-1, 007c, and *Ruminococcus champanellensis*18P13), presumably because RefSeq uses stringent criteria for inclusion of genomes in their database and these genomes were of insufficient quality. These species genomes were manually added into our analysis for completeness’ sake. A custom python (v. 3.11.5) script was used to analyze the domain composition in the scaffoldins and dockerin-fused proteins in the 37 bacterial species that produce cellulosomes. The domain identifiers used for this analysis are listed in Supplementary Table S5.

### AlphaFold2 analysis to identify cohesin-containing proteins

We observed that 34 protein sequences (from 3 organisms: *R. flavefaciens* 17, *R. flavefaciens* 007c, and *R. champanellensis*) are reported in the literature to possess sequence homology to cohesin domains but we could not identify them as such using the HMM profiles provided by InterProScan (Dassa et al., 2014). Similarly, our analysis showed significantly lower numbers of scaffoldin proteins for additional *R. flavefaciens* strains (AE3010, SAb67, YL228) that we suspected also encode cohesin domains that are too divergent to be identified by the HMM profiles. Recognizing that structure is better conserved than sequence, we used AlphaFold2 (Jumper et al., 2021) to build atomic models for any protein across these six organisms that contained at least one of the following domains: Coh1 (cd08548), Coh2 (cd08547), Coh3 (cd08759), general Coh (PF00963), CBM2/3 Superfamily (IPR008965), CATH3D entry (G3DSA:2.60.40.680). This resulted in the predictions of 90 putative cohesin-containing proteins. We requested three models to be built for each sequence and used only the model with highest pLDDT score for further structural analysis. For multi-domain proteins, we divided the model into individual domains using the program UniDoc (Zhu et al., 2023), producing 311 domains. The structure of each domain was compared to four reference models: Coh1 (PDB:1OHZ), Coh2 (PDB:1TYJ), Coh3 (PDB:2FZ9), and CBM3 (PDB:6UFW). Structural comparisons were performed with the program TMalign (Dong et al., 2018). TM-scores for all comparisons were normalized to 140 residues to simplify comparison. Query domains with TM-scores greater than 0.50 were considered as matches to the reference model. If a domain scored higher than 0.50 for multiple reference models, then we attribute the domain identity to match the highest scoring reference model.

## Data Availability

Information for existing publicly accessible datasets is contained within the article. The genome accession numbers presented in this study can be found in supplementary table 4 and online through the RefSeq or GenBank repositories. The original InterProScan analaysis will be provided upon request. Any further inquiries can be directed to the corresponding author(s).
