# Supplementary material for: A genomic analysis reveals the diversity of cellulosome displaying bacteria
Source: Front Microbiol. 2024 Oct 30;15:1473396. doi: 10.3389/fmicb.2024.1473396 (PMC11557425; doi:10.3389/fmicb.2024.1473396)
Supplement: Supplementary file 1 [file Data_Sheet_1.PDF]

SUPPLEMENTARY FIGURES

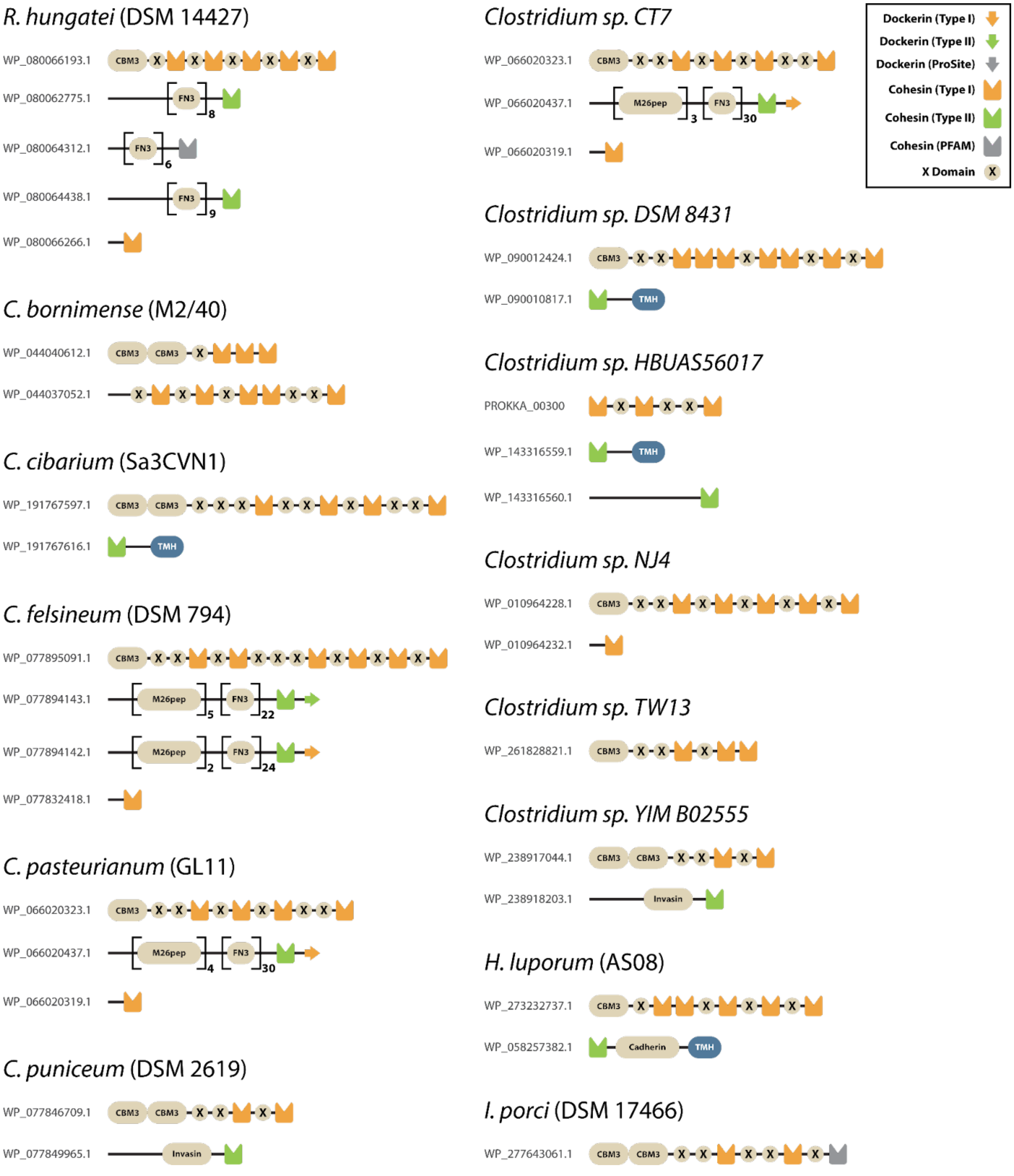

Figure S1. Schematic representation of unreported scaffoldins from known cellulosome producers. Cartoon representation showing the cohesin-containing proteins present within simple cellulosome producing organisms whose scaffoldin compositions have not been reported previously. Novel organisms identified in this study include: *Clostridium cibarium* (Sa3CVN1), *Clostridium pasteurianum* (GL11) ,*Clostridium sp. CT7*, *Clostridium sp. DSM 8431*, *Clostridium sp. HBUAS56017*, *Clostridium sp. NJ4*, *Clostridium sp. TW13*, *Clostridium sp. YIM B02555*, *Inconstantimicrobium porci* (DSM 17466). Domains are

colored according to the upper right key with additional domains including: FN3 (beige)- Fibronectin type III (SM00060), CBM3 (beige)- Carbohydrate binding module family 3 (PF00942), M26pep (beige) - M26 Metallo-endopeptidase (PF05342), TMH (navy) - Transmembrane Helix, Invasin (beige)- Invasin/intimin cell-adhesion fragment (SSF49373), and Cadherin (beige) - Cadherin-like (PF12733).

SUPPLEMENTARY FIGURES

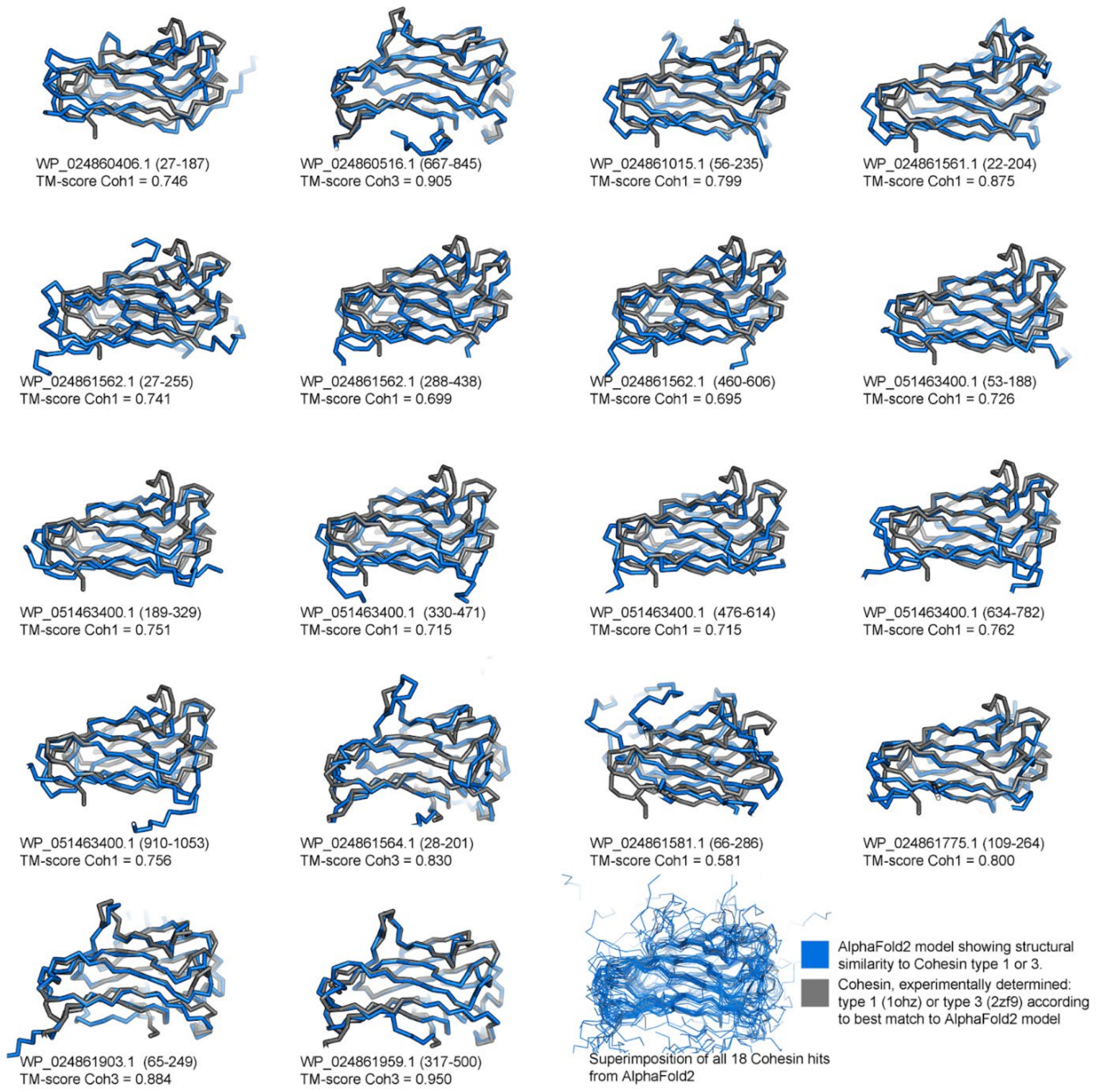

Figure S2: Superposition of AlphaFold2 identified cohesin domains within *Ruminococcus flavefaciens* (AE3010) Eighteen cohesin domains were identified by AlphaFold2 in *Ruminococcus flaefaciens* (AE3010). The blue chain represents the predicted AlphaFold2 model, and the gray chain shows either an experimentally determined Coh1 (PDB: 1OHZ) or Coh3 (PDB: 2FZ9). Fourteen Coh1 domains and four Coh3 domains were captured with a TM-score above 0.5 to either representative structures of Coh1 (PDB: 1OHZ) or Coh3 (PDB: 2FZ9), respectively. The last panel is a superposition of all 18 hits. Six domains had been identified using any of the following cohesin-type identifiers (coh1 (cd08548), coh2 (cd08547), coh3 (cd08759), coh (PF00963)). A total of 18 domains were identified by AlphaFold2 to be cohesin modules.
